# Small Extracellular Vesicle-Derived microRNAs Stratify Prostate Cancer Patients According to Gleason Score, Race and Associate with Survival of African American and Caucasian Men

**DOI:** 10.3390/cancers13205236

**Published:** 2021-10-19

**Authors:** Hamdy E. A. Ali, Mohamed S. A. Gaballah, Rofaida Gaballa, Shahenda Mahgoub, Zeinab A. Hassan, Eman A. Toraih, Bettina F. Drake, Zakaria Y. Abd Elmageed

**Affiliations:** 1 Department of Pharmaceutical Sciences, Rangel College of Pharmacy, Texas A&M University, College Station, TX 77843, USA; haali@tamu.edu (H.E.A.A.); mohamed.gaballah@pharm.helwan.edu.eg (M.S.A.G.); Rofayda011142@pharm.bsu.edu.eg (R.G.); 2 Department of Radiobiological Applications, Nuclear Research Center, Atomic Energy Authority, Cairo 13759, Egypt; 3 Department of Biochemistry and Molecular Biology, Faculty of Pharmacy, Helwan University, Cairo 11795, Egypt; Shahenda.mahgoub@pharm.helwan.edu.eg (S.M.); zihassan2014@gmail.com (Z.A.H.); 4 Department of Surgery, Tulane University School of Medicine, 1430 Tulane Avenue, New Orleans, LA 70112, USA; etoraih@tulane.edu; 5 Division of Public Health Sciences, School of Medicine, Washington University, Saint Louis, MO 63110, USA; drakeb@wustl.edu; 6 Department of Pharmacology, Edward Via College of Osteopathic Medicine, University of Louisiana at Monroe, Monroe, LA 71203, USA

**Keywords:** prostate cancer, small extracellular vesicle-associated microRNAs, biomarker, African American, clinical outcomes

## Abstract

**Simple Summary:**

Novel biomarkers are needed to guide prognosis and treatment of aggressive forms of prostate cancer (PCa). In this study, small extracellular vesicles (sEVs)-derived microRNAs (miRs) are used to predict aggressive phenotypes and ancestral background of PCa patients. Two cohorts was used to study the diagnostic and prognostic utility of sEV-associated miRs in predicting aggressive forms of PCa in African American (AA) and Caucasian (CA) men. In training cohort, miR profiling was performed and top-ranked sEV-associated miRs were then validated in two independent confirmatory cohorts comprising 150 plasma samples. Results revealed that sEV-associated miR-6068 and miR-1915-3p were enriched in PCa patients compared to healthy subjects. sEV-associated miR-6716-5p and miR-3692-3p distinguished AA from CA men and low from high Gleason score. However, miR-1915-3p was the only studied miR associated with longer recurrence-free survival as independent prognostic marker.

**Abstract:**

The utility of small extracellular vesicles (sEVs)-derived microRNAs (miRs) to segregate prostate cancer (PCa) patients according to tumor aggressiveness and ancestral background has not been fully investigated. Thus, we aimed to determine the diagnostic and prognostic utility of sEV-associated miRs in identifying aggressive PCa in African American (AA) and Caucasian (CA) men. Using a training cohort, miR profiling was performed on sEVs isolated from plasma of PCa patients. Top-ranked sEV-associated miRs were then validated in 150 plasma samples (75 AA and 75 CA) collected from two independent cohorts; NIH (*n* = 90) and Washington University (*n* = 60) cohorts. Receiver operating characteristic (ROC) curve, Kaplan–Meier and Cox proportional hazards regression were used to assess these miRs as clinical biomarkers. Among nine top-ranked sEV-associated miRs, miR-6068 and miR-1915-3p were enriched in sEVs collected from PCa patients compared to healthy volunteers. Moreover, miR-6716-5p and miR-3692-3p segregated AA from CA men and low from high Gleason score (GS), respectively. Upregulation of sEV-associated miR-1915-3p, miR-3692-3p and miR-5001-5p was associated with improved survival time, and only miR-1915-3p was associated with longer recurrence-free survival (RFS) as an independent prognostic marker. Taken together, we identified novel sEV-associated miRs that can differentiate PCa patients from normal, AA from CA and high from low GS and predicts RFS.

## 1. Introduction

Prostate cancer (PCa) is the second leading cause of death in elderly men living in the United States. It was reported that more than 248,530 cases will be diagnosed and 34,130 deaths are estimated in 2021 [1]. Reliable molecular biomarkers can serve as accurate tools for monitoring the clinical progression of the disease and the overall survival of cancer patients. Because PCa is a biologically and clinically heterogeneous disease, none of the current PCa molecular biomarkers are ideal to contend with the wide variety of human specimens, states of the disease and other clinical outcomes [2]. Although prostate-specific antigen (PSA) is a routine marker used for diagnosis and prognosis of PCa, its sensitivity is compromised by false-positive results, which might lead to overtreatment of indolent disease [3] and mislead treatment decisions at relapse [4]. Therefore, the development of new strategies for early detection and prognosis of PCa is an unmet clinical need. The mortality rate of PCa in African Americans (AAs) is twice as high as that of Caucasian American (CA) men [5]. It was reported that AA men have a bigger PCa tumor size than CA men and that tumor cells are more likely to transform from dormant to aggressive cells [6]. At high grades of the disease, PSA level and biochemical recurrence are higher in AA men with PCa [7]. Although the gene expression profiling approach has been used to segregate clinical outcomes of the disease, the ability of differentially enriched microRNAs (miRs) in the circulating small extracellular vesicles to segregate PCa patients based on their races/ethnicities, and aggressive forms of the tumor have not been fully investigated.

Small extracellular vesicles (sEVs) are tiny cell-derived extracellular bodies (<200 nm in diameter) of endosomal origin released by almost all cells into body fluids to promote cell–cell communications [8]. sEVs act as targeted delivery devices for various biological materials such as microRNAs (miRs), mRNAs, lipids, DNA, proteins and metabolites to recipient cells [9,10]. sEVs are formed by fusing multivesicular bodies into the cell membrane, and therefore the molecular content of sEVs is mainly dependent on their parental cells. miRs are important regulators of a wide array of normal and pathological cellular processes, including tumor progression and metastasis [11]. As a result, identification of disease-specific sEV-associated miRs will facilitate the use of these vesicles as a source of new biomarkers in the diagnosis, prognosis and surveillance of PCa patients. For example, a large-scale study reported the use of circulating miR-17-3p and miR-1185-3p to discriminate PCa patients from those who had negative biopsies with high accuracy [12].

Herein, we report that sEV-associated miR-6068 and miR-1915-3p can discriminate PCa patients from age-matched healthy volunteers. Intriguingly, miR-6716-5p was able to distinguish AA from CA PCa patients, while miR-3692-3p and miR-1915-3p were differentially enriched in sEVs at different Gleason scores. The enriched miR-1915-3p, miR-3692-3p and miR-5001-5p in sEVs were associated with the improved survival time. Multivariate Cox regression analysis revealed that miR-1915-3p was an independent prognostic factor for overall survival (OS) and recurrence-free survival (RFS).

## 2. Results

### 2.1. Characterization of Small Extracellular Vesicles

**Figure 1:**
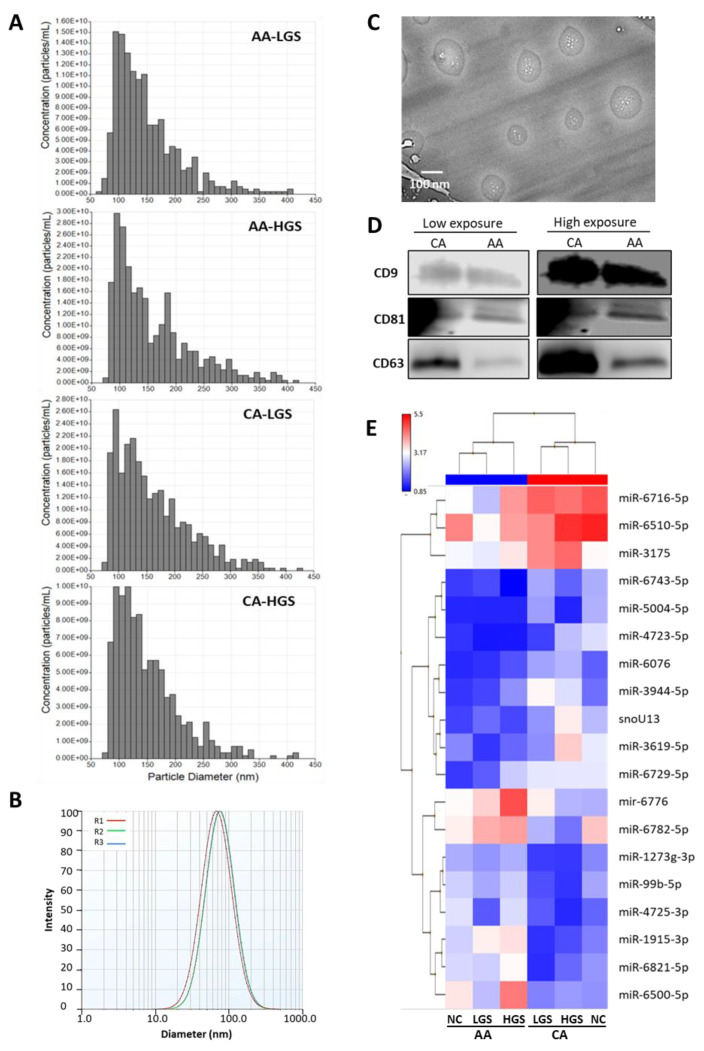
Characterization of small extracellular vesicles (sEVs) isolated from the plasma of PCa patients and miRs profiling of sEVs. (**A**): Characterization of sEVs isolated from blood samples of AA and CA PCa patients. The sizes of sEVs was measured by the qNano method at LGS and HGS of AA and CA patients. (**B**): Representative graph showing the size of sEVs measured by ZetaPALS analysis. (**C**): Cryo-TEM micrograph showing the diameter of sEVs. Scale bar is 100 nm. (**D**): Expression of surface protein markers (CD9, CD81 and CD63) of sEVs isolated from AA and CA PCa samples detected by Western blot analysis. Complete blots are available in Figure S1. (**E**): Microarray analysis of sEV-associated miRs collected from plasma of AA and CA PCa patients in addition to age- and race-matched normal subjects. Representative heatmap showing the upregulated (red) and downregulated (blue) sEV-associated miRs in AA versus CA PCa normalized to healthy volunteers. AA: African American; CA: Caucasian American; NC: Normal control subjects; HGS: high Gleason score; LGS: low GS; R1-3: number of repeats of sEVs.

Before we initiated our study, which aimed to underpin the clinical utility of sEV-associated miRs in stratification of AA and CA men with PCa, the sizes of sEVs were verified by nano method, ZetaPals Zeta Potential Analyzer (Brookhaven Instruments Corporation, Holtsville, NY, USA) and TECNAI Ti Cryo-TEM (Field Electron and Ion Company, Hillsboro, OR, USA) in the two cohorts, and the results show that the isolated vesicles had an average diameter of <200 nm, which fits within the regular range of sEVs (Figure 1A–C) as reported in MISEV-2018 guidelines [8]. The average size of sEVs measured by qNano analysis ranged between 145 to 162 nm in diameter (Table S3). The average diameter of sEVs measured by ZetaPals was 139 nm, while Cryo-TEM results show the size range of the vesicles was 90–166 nm. The purity of sEVs was also confirmed by surface protein markers using immunoblot analysis as previously reported [13,14,15]. Total protein lysates of sEVs were immunoblotted with antibodies raised against CD9, CD81 and CD63, which produced positive signals in sEVs isolated from plasma samples of AA and CA PCa patients (Figure 1D).

### 2.2. sEV-Associated miR Profiling

To identify the differentially expressed miRs in sEVs, miR profiling was performed. All mature and pre-mature miRs covered by the Affymetrix miRNA Array v. 4.0 (Thermo Fisher Scientific, Waltham, MA, USA) were compared for RNA pools of sEVs isolated from plasma of PCa patients and normal individuals represented by fold change and *p*-value. Initially, when a fold change (FC) of 1.5 and *p*-value of <0.05 calculated by ANOVA were applied, 185 differentially packaged miRs were identified in sEVs of PCa in comparison to normal blood, 178 differentially packaged miRs in sEVs collected from the blood of PCa patients at high Gleason score (HGS) versus low GS (LGS), and 101 differentially packaged miRs in sEVs of AA versus CA of PCa patients (Table S4). We then used FC of 2.0 to improve the biological significance of the selected miRs. By applying the new FC of 2.0, 85 miRs were differentially packaged in sEVs of PCa versus normal subjects, 43 miRs differentially packaged in sEVs of HGS versus LGS, and 19 miRs differentially packaged in sEVs of AA versus CA men (Table 1). As shown in Table 1 and Figure 1E, hierarchical clustering of differentially packaged miRs are clearly separated samples procured from AA and CA men into two main clusters. Nineteen miRs met these criteria when the race was considered, where 8 miRs were upregulated and 11 miRs were downregulated. Hierarchical clustering was also able to stratify miRs according to Gleason score regardless of the race, where 43 miRs were differentially packaged in HGS when compared to LGS. Approximately 85 miRs were differentially enriched in sEVs of PCa patients compared to their normal counterparts regardless the race and GS.To further enhance our selection criteria for the biological significance and compensate the higher FDR value, the top dysregulated miRs that possess >2.0 FC and *p*-value of <0.005 were only selected for q-PCR validation. 

### 2.3. Pathway Prediction of sEV-Associated miRs

Figure S2 demonstrates the pathway analysis of the target genes of the differentially packaged miRs. miR-5001 and miR-5189 are enriched in axonal guidance, endocytosis, cell adhesions, RAS and PI3K signaling, and proteoglycans in cancer. miR-1915-3p, miR-3944-5p and miR-6716-5p are linked to ECM-receptor interaction, fatty acid biosynthesis and mucin-type o-glycan biosynthesis.

### 2.4. Differential Expression of sEV-Associated miRs in PCa Patients Compared to Normal Individuals in the Confirmatory Cohorts

To confirm the miR profiling results, qPCR analysis was performed to examine the expression of top listed miRs and tested in the two cohorts recruited by the University of Washington (W cohort), and the National Cancer Institute, NIH (NIH cohort). The ΔCT was calculated for sEV-associated miRs derived from normal and tumor subjects and compared by Student *t*-test. The ΔCT results showed that miR-1915-3p was the only miR enriched in sEVs of PCa in comparison with normal sEVs (*p* = 0.001), while miR-6068, miR-3692-3p, miR-3939, miR-5189-5p and miR-6716-5p were enriched in sEVs of normal compared to PCa sEVs (*p* < 0.004). However, miR-3201, 3944-5p and miR-5001-5p showed no significant changes among the two groups (Table 2).

### 2.5. sEV-Associated miRs Discriminating PCa Patients from Normal Subjects with High Accuracy

For those miRs that have shown differential expression between PCa and healthy control groups, the sensitivity, specificity and area under curve (AUC) were calculated. The highest sensitivity (84 and 76%) and AUC (0.809 and 0.809) among the studied induvial miRs was recorded for miR-3939 and miR-6068, respectively. When miR-1915-3p was combined with miR-6716-5p, the miR accuracy slightly increased to 0.833. Furthermore, when the five miRs are incorporated, the AUC value was significantly improved to 0.904 (*p* < 0.0001) as depicted in Table 3.

### 2.6. Differential Packaging of sEV-Associated miRs in AA versus CA PCa Patients

When the race was considered in the Washington (W) cohort, three miRs were enriched in sEVs collected from the plasma of AA compared to CA PCa men. Statistical comparison between AA and CA was calculated by Student *t*-test. As presented in Table 4, these miRs are miR-3939 (*p* = 0.005), miR-6716-5p (*p* = 0.007) and miR-5189-5p (*p* = 0.033). The rest of miRs failed to show any significant differences between the two groups. In the NIH cohort, only miR-3692-3p and miR-6716-5p were highly enriched in sEVs of AA versus CA sEVs (*p* < 0.001 and *p* = 0.003, respectively). However, miR-3939, miR-5189-5p, miR-1915-3p, miR-5001-5p, miR-3201 and miR-3944-5p did not achieve statistical significance (Table 4). Overall, miR-6716-5p is the only upregulated miR in sEVs collected from AA when compared to those of CA PCa patients in discovery and confirmatory cohorts.

### 2.7. sEV-Associated miRs Discriminating AA PCa Patients from CA PCa Patients

In the W cohort, the highest accuracy (AUC = 0.705) was detected by miR-3939 (Table 3). When miR-3939 and miR-6716-5p are combined, the two miRs achieved the AUC value of 0.754. Interestingly, when miR-3939, miR-5189-5p and miR-6716-5p were collectively combined, the accuracy was slightly improved (AUC = 0.761). Compared with the W cohort, two individual miRs—miR-6716-5p and miR-3692-3p from the NIH cohort had AUC values of 0.706 and 0.665, respectively, and when they are combined, the AUC value improved to 0.752 (Table 3).

### 2.8. Differential Packaging of sEV-Associated miRs in PCa Patients Based on Gleason Score (GS)

The plasma samples received from W-cohort had only two categories of GSs—high GS (HGS) and low GS (LGS)—which were compared using Student’s *t*-test. As shown in Table 4, miR-1915-3p and miR-3692-3p were enriched in sEVs of PCa patients based on GS, while the other six miRs did not show any significance. In the NIH cohort, three categories of GS were provided—HGS, GS at 7 and LGS—and one-way ANOVA was used to compare the three groups followed by post hoc Tuckey test.

One-way ANOVA analysis showed that miR-1915-3p, miR-3692-3p, miR-5001-5p and miR-6068 exhibited a differential packaging at different GS (*p* = 0.008, *p* = 0.010, *p* = 0.032 and 0.015, respectively). Using post hoc Tuckey test, the sEVs level of miR-1915-3p in patients with LGS was significantly lower than that in patients at GS7 (*p* = 0.009) or HGS (*p* = 0.046). miR-3692-3p was more enriched in sEVs collected from patients at LGS than that of HGS (*p* = 0.011). The level of miR-5001-5p was higher in patients at GS7 compared to LGS (*p* = 0.024) and was not able to achieve the statistical significance when LGS was compared to HGS (*p* = 0.356). miR-6068 was more enriched in LGS compared to either GS7 (*p* = 0.030) or HGS (0.031). Collectively, miR-3692-3p was confirmed that it is differentially enriched in sEVs at different GS considering training cohorts and the other two confirmatory cohorts.

### 2.9. The Ability of sEV-Associated miRs to Stratify PCa Patients According to Their Gleason Scores

To stratify PCa patients according to their GS in the W cohort, individual miR-1915-3p and miR-3692-3p exhibited AUC values of 0.662 (sensitivity = 73%) and 0.661 (sensitivity = 61%), respectively. When the two miRs were combined, the AUC value reached 0.683 (Table 3). In the NIH cohort, the accuracy of using individual miR-1915-3p, miR-3692-3p and miR-5001-5p to discriminate patients based on their GS was 0.700, 0.661 and 0.697, respectively. Interestingly, when the three miRs were combined, the AUC value was improved to 0.818 (Table 3).

As shown in Table S5, when the GS and race are considered in the W-cohort, miR-1915-3p (AUC = 0.847) and miR-3692 (AUC = 0.860) discriminated AA-HGS from AA-LGS with high accuracy as individual predictors. The combination of the two miRs slightly improved the AUC value to 0.872. In addition, AA-LGS can be differentiated from CA-LGS and CA-HGS with high accuracy by blotting ROC curves, and the two miR combinations improved the AUC to 0.888 and 0.883, respectively. In the NIH cohort, AA-HGS was able to be differentiated from AA-LGS with high accuracy, and when these miRs were combined, the AUC value reached 1 (Table S6).

### 2.10. sEV-Associated miRs as Potential Prognostic Markers for PCa

**Figure 2:**
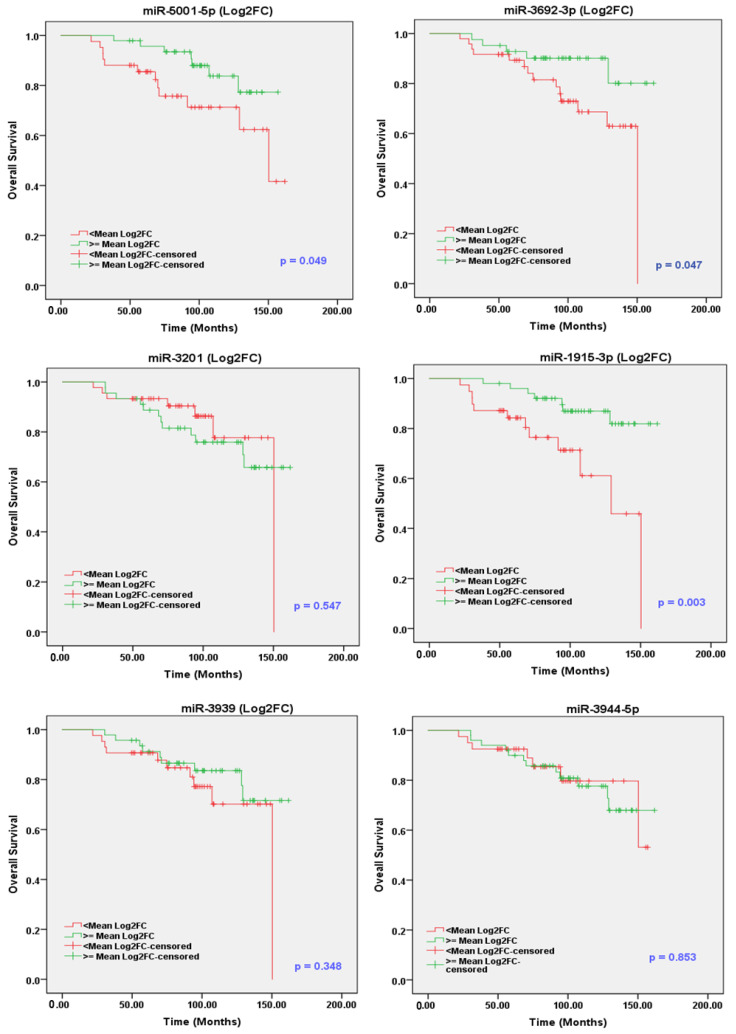
Overall survival analysis for PCa patients expressing high and low levels of sEV-associated miR-1915-3p, miR-3201, miR-3939, miR-3944-5p, miR-miR-3692-3p and miR-5001-5p in the NIH cohort. Kaplan–Meier analysis showing sEVs enrichment of miR-1915-3p, miR-3692-3p and miR-5001-5p is associated with better survival in PCa. The *p*-value was calculated by the log-rank test of the Kaplan–Meier curve.

**Figure 3:**
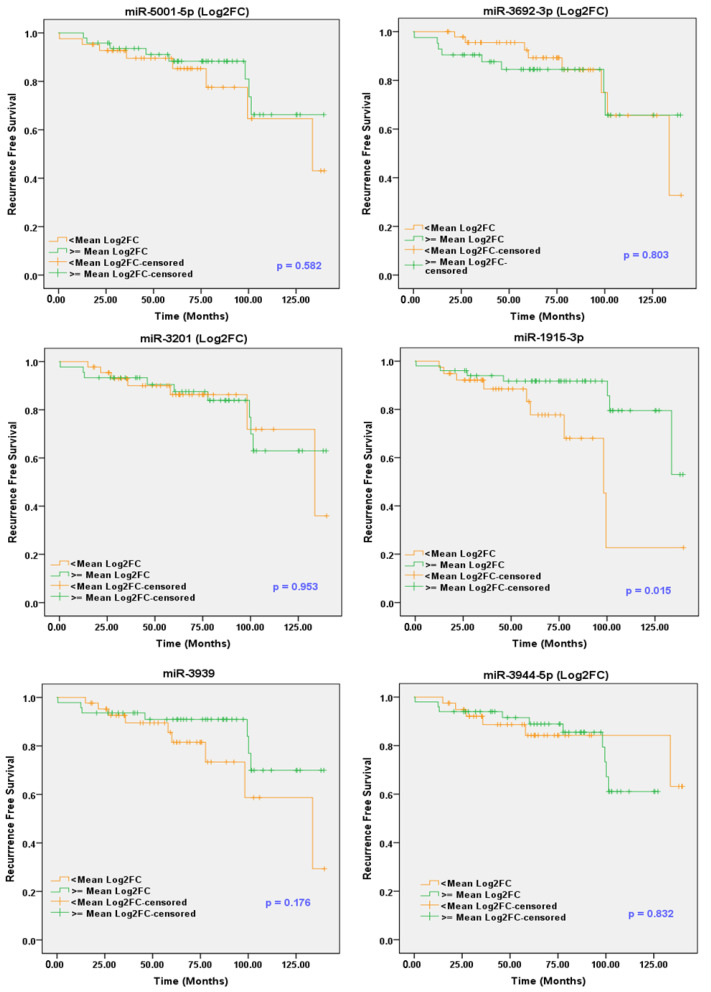
Recurrence-free survival analysis for PCa patients expressing high and low levels of sEV-associated miR-1915-3p, miR-3201, miR-3939, miR-3944-5p, miR-miR-3692-3p and miR-5001-5p in NIH cohort. Kaplan–Meier analysis showing that PCa patients had better recurrence-free survival with sEVs enrichment of miR-1915-3p. The *p*-value was calculated by the log-rank test of the Kaplan–Meier curve.

Considering the mean value as cutoff, the expression value of each miR was expressed as Log2FC dichotomized into two categories: a low-expression group (<mean) and a high-expression group (≥mean). Median overall survival (OS) and recurrence-free survival (RFS) for the two subgroups were calculated by the Kaplan–Meier method (Figure 2 and Figure 3). The survival curves for the two subgroups were compared using the log-rank test. In addition, the clinicopathological features, including race, age, Gleason score, stage, PSA, and smoker status, were analyzed with the survival data.

Interestingly, the OS analysis revealed that the high levels of sEV-associated miR-1915-3p, miR-3692-3p and miR-5001-5p are associated with the improved survival time, with log-rank *p*-values of 0.003, 0.047 and 0.049, respectively (Figure 2). Moreover, the upregulated miR-1915-3p demonstrates a significant association with longer RFS (log-rank *p* = 0.015), as shown in Figure 3. There was no significant relationship between the expression of other miRs and OS or RFS. As depicted in Table 5 and Table 6, sEV-associated miRs that achieve statistical significance in the univariate analysis and/or Kaplan–Meier (*p* ≤ 0.15) are included in the multivariate cox regression proportional hazard analysis considering the demographic and clinical covariates of PCa in this model. These parameters included age, race, GS, PSA, tumor stage and smoking status (Figures S3 and S4). To evaluate their independent prognostic utility, the selected sEV-associated miRs were initially included in the multivariate cox regression model. Then, we applied the backward elimination method to drop the non-significant covariates and keep only the significant ones. The multivariate Cox regression analysis revealed that miR-1915-3p was able to keep its significance as an independent prognostic factor for both OS and RFS of PCa patients.

## 3. Discussion

sEV-associated miRs isolated from the plasma of PCa patients have emerged as reliable noninvasive diagnostic and prognostic markers. However, PCa is a heterogeneous disease, and the discovery of surrogate markers used for diagnosis and prediction of clinical outcomes of patients with PCa is an unmet clinical need. In this study, we report that among the nine sEV-associated miRs studied, miR-6068 and miR-1915-3p were the only miRs enriched in sEVs collected from PCa in comparison to age-matched healthy volunteers in all cohorts. When the race was adjusted, miR-6716-5p is the only upregulated miR in sEVs collected from AA when compared to those of CA PCa patients in discovery and confirmatory cohorts. When the tumor stage was considered, miR-3692-3p and miR-1915-3p were differentially enriched in sEVs at different Gleason scores. The high levels of sEV-associated miR-1915-3p, miR-3692-3p and miR-5001-5p are associated with the improved survival time, and only miR-1915-3p demonstrates a significant association with longer RFS. The multivariate Cox regression analysis revealed that miR-1915-3p was able to maintain its significance as an independent prognostic factor for the OS and RFS of PCa patients.

Our data suggested for the first time that sEV-associated miR-6068 can differentiate between PCa patients and normal individuals. In other cancer types, miR-6068 was reported to be upregulated in endometrial cancer tissues regardless of lymph node status [16] and tissue and plasma of lung squamous cell carcinoma patients [17]. However, it was downregulated in sEVs isolated from the blood of colorectal cancer [18] and after treatment of non-small cell lung cancer cells with piperlongumine [19]. We evaluated the unreported miR-6716-5p in PCa, and it was found to differentiate between AA and CA men. In the same context, other studies showed that a specific set of miRs are differentially enriched in sEVs versus whole plasma collected from blood samples of PCa patients compared to benign prostatic hyperplasia [20]. In a study conducted on tissues collected from colorectal cancer patients, miR-6716-5p was upregulated, promoted cell migration and invasion and was associated with inferior clinical outcomes [21]. Other miRs such as miR-4288 were also reported to distinguish CA from AA men with PCa [22]. This miR is associated with high GS and PSA and therefore predicts aggressive tumor phenotypes. The same group reported that the downregulation of miR-3622b in PCa tissues upregulates EGFR, a condition that may lead to poor prognosis [23]. Since the incidence and mortality rates of PCa are higher in AA than in CA men, miR-6716-5p may be used for prediction of cancer aggressiveness in AA men but needs further validation.

Although our group is the first one to report the prognostic role of sEV-associated miR-1915-3p in PCa, other research groups reported its free-circulated form as a diagnostic marker and predictor of patients’ clinical outcomes in different malignancies. Though the role of miR-1915-3p has not been explored in PCa, its level was upregulated in breast cancer patients and has been suggested for use as a diagnostic biomarker [24]. In the same study, upregulated miR-1915 was positively correlated with lymph node metastasis, and its ectopic expression increased the activity of ERK1/2 through repression of DUSP3. On the contrary, this miR was downregulated in MCF7 cells, and its ectopic expression resulted in retardation of cell growth and migration through repression of SETD1A [25]. This miR was able to stratify patients with diffuse glioma compared to individuals who had other CNS-related diseases and healthy volunteers [26]. However, its downregulation was reported in gastric cancer and was correlated with lymph node metastasis and overall survival [27]. Upregulation of miR-1915-3p is associated with infiltrative growth of follicular variant of papillary thyroid carcinomas [28], oxidative stress responses and antiapoptotic pathway in hepatocellular carcinoma [29], and immune regulation and cell cycle in bone marrow mesenchymal stromal cells collected from elder donors [30]. In lung cancer cells, miR-1915 is suggested as an antiapoptotic non-coding RNA by targeting DRG2/PBX2 [31]. After inducing DNA damage by adriamycin, Bcl-2 expression was negatively regulated by p53 through miR-1915 axis in colorectal carcinoma cells [32]. It was reported that miR-17/miR-192 and miR-181a in plasma collected from patients are used as a specific panel for the prediction of aggressive forms of PCa [33]. The same research group could not identify any miR that can differentiate between AA and CA patients and attributed it to the smaller size of CA samples. Regarding miR-5001-5p, the miR was upregulated in endometrial cancer, but its expression is not associated with positive lymph node metastasis [16]. In our study, a balanced pair of samples were included in each of the discovery and confirmatory cohorts to signify any expression differences.

We validated two miRs, 3692-3p and 1915-3p, which discriminate between low and high GS. We provide the first report which demonstrates the prognostic role of sEV-associated miR-3692-3p in PCa staging. However, other few studies indicate that miR-3692-3p was upregulated in blood collected from patients with non-small cell lung carcinoma as compared to healthy counterparts [34]. Functional polymorphisms identified in the 3′-UTR of B7/CD28 genes dysregulate the miR-3692-3p/B7/CD28 axis in colorectal cancer [35]. This suggests the expected role of miR-3692-3p in the progression of PCa, but more investigations are warranted. It was reported that miR-3692-3p is upregulated in urine collected from PCa patients who had a BCR and at a higher stage of the disease [36]. Other reports highlighted different miRs that are associated with higher pathologic grade, positive lymph nodes and distant metastasis, and poor prognosis of PCa [37]. In 2019, Richardsen et al. reported that the high level of miR-141 in PCa tissue specimens is correlated with aggressive clinical outcomes including GS [38]. Thus, our findings corroborated other studies that suggest miR-3692-3p and 1915-3p as promising candidates for discriminate between different pathological stages of the tumor.

The inconsistency of miRs expression among different studies is the main obstacle that hampered the clinical utilities of miRs as diagnostic and/or prognostic biomarkers. These include but are not limited to a lack of standardized analytical and detection methods, tumor heterogeneities, relatively low numbers of cases, poor representative samples and misleading interpretations [39]. However, our study used a discovery cohort followed by two independent NIH and Washington cohorts, which comprise paired plasma samples collected from AA and CA men. This should increase the power of statistics and provide more evidence for validating the collected data. The purity of sEVs was confirmed by qNano, ZetaPals Zeta Potential analysis, Cryo-TEM and sEVs surface protein markers as previously reported [13,40]. Future studies are needed to validate these sEV-associated miRs in a large number of samples using a multi-institutional study and correlate this panel with other reported genomic and non-genomic differences in AA and CA men with PCa.

## 4. Materials and Methods

### 4.1. Clinical Samples

Written informed consent was obtained from patients prior to initiating the study. The current study was conducted in accordance with the guidelines of protocols approved by the Institutional Review Board (IRB) from the Texas A&M Health Science Center (IRB#2017-0190M), College Station, TX, USA, Edward Via College of Osteopathic Medicine, Monroe, LA (IRB#2020-036), Washington University School of Medicine, St. Louis, MO, USA and the National Cancer Institute, NIH, Bethesda, MD, USA. The discovery cohort comprised 24 PCa plasma samples: 12 AA and 12 CA. The confirmatory cohort comprised 150 plasma samples collected from PCa patients (75 AA and 75 CA) at different Gleason scores (GS) obtained from the NIH (90 samples) and Washington University through Cooperative Human Tissue Network (CHTN, 60 samples) and stored at −80 °C till used. All available clinical information was obtained from NCI, NIH and CHTN/Prostate Cancer Biorepository Network (PCBN). In addition, thirty plasma samples collected from age- and race-matched healthy individuals were obtained from BioIVT (Westbury, NY, USA) and considered as PCa-free (healthy) controls.

### 4.2. Isolation and Characterization of sEVs, and Extraction of sEV-Associated RNA

sEVs were isolated from plasma of AA and CA PCa patients using ExoQuick^®^ UTRA EV isolation kit (System Biosciences, Palo Alto, CA, USA) following the manufacturer’s instructions. The size and concentration of sEVs were determined by Tunable Resistive Pulse Sensing (TRPS) technique using qNano following the manufacturer’s instructions (Izon Science Ltd., Cambridge, MA, USA) and as reported [41]. Briefly, after isolation of sEVs from the plasma of PCa patients, sEVs suspension was diluted 1:2 (*v*/*v*) in PBS and subjected to qNano analysis using the NP100 nanopores. The size and concentration of sEVs were recorded using Izon control Suite Software. In another experiment, the size of sEVs was also validated in the two cohorts using ZetaPals Zeta Potential Analyzer, Cryo-Transmission Electron Microscope (TEM) and surface protein markers by Western blotting as we reported [13,42]. sEV-associated RNA was then isolated using miRCURY RNA isolation Kit Bio-fluids (Exiqon) according to manufacturer’s protocol. For miR profiling only, six RNA pools were prepared as follows: AA high Gleason score (HGS), AA low GS (LGS), CA-HGS, CA-LGS, normal AA and normal CA. Each pool sample was age- and Gleason-score-matched. For characterization and validation experiments, RNA was extracted from sEVs derived from individual plasma samples without pooling for performing sEVs characterization and sEV-associated miRs expression using quantitative Real-Time-PCR (qPCR) analysis.

### 4.3. Western Blot Analysis

About 200 µL of plasma collected from 174 PCa and 30 healthy individuals was centrifuged at 3000× *g* for 15 min to remove cellular debris, and the supernatant was transferred to a new tube. The supernatant was passed through a 0.22 µm filter unit, mixed with 2 µL of 500 U/mL Thrombin and incubated for 5 min (Qiagen, Germantown, MD, USA). The samples were centrifuged at 10,000× *g* for 5 min, and the supernatant was then collected. The purification of sEVs was performed by ExoQuick^®^ UTRA EV isolation kit (System Biosciences, Palo Alto, CA, USA) following the standard protocol. Briefly, 200 µL plasma spun at 3000× *g* for 15 min, and the supernatant was centrifuged again at 12,000× *g* for 15 min. About 67 µL of ExoQuik reagent was added to the supernatant, mixed and kept at 4 °C for 12–16 h. The mixture was centrifuged at 3000× *g* at 4 °C for 10 min. The sEVs pellets were resuspended, and column purification was performed. RIPA buffer was added to the sEVs pellets and the protein lysate was resolved on 4–20% SDS-PAGE electrophoresis.

### 4.4. microRNA Profiling of sEVs (Discovery Cohort)

miR profiling was carried out in the Non-coding RNA Core (MD Anderson Cancer Center, Houston, TX, USA) using Affymetrix platform. RNA quality and quantity were assessed using Agilent 2100 BioAnalyzer (Agilent Technologies, Santa Clara, CA, USA). Total plasma sEV-associated RNA of the six pools (Table S1) of AA and CA PCa patients, as well as the two pools of the healthy controls (AA and CA), was then profiled using an Affymetrix GeneChip miRNA Array v. 4.0 (Affymetrix, Santa Clara, CA, USA). An average of 1.0 µg of total RNA was labeled, hybridized, washed and scanned according to the manufacturer’s protocol. All mature miR, as well as pre-miRs covered by the Affymetrix miRNA Array v. 4.0, were compared for all plasma sEV-associated RNA pools represented by fold change and *p*-value. The cutoff value was 2.0 for fold change with a *p*-value of less than 0.05 calculated by ANOVA.

### 4.5. Validation of the Differentially Packaged miRs by qPCR (Confirmatory Cohorts)

Differentially packaged miRs into sEVs from miR profiling results were then selected and validated by qPCR using 150 PCa plasma samples provided by the NIH and Washington University and considered as validation cohorts. Due to the high FDR *p*-value from the microarray study, the top candidate microRNAs (miRs) that showed differential packaging in the compared groups were then individually validated in triplicates by qPCR in the sEV-associated RNA isolated from two different cohorts of PCa patients as well as an age-matched control group. The available clinical data of the two confirmatory cohorts are presented in Table S2. The analyzed data was presented as Log2FC, and significance was considered at *p* < 0.05. The *p*-value was calculated by Student *t*-test or ANOVA according to the number of compared groups.

### 4.6. Pathway Prediction for sEV-Associated miRs

The heatmap and clustering of predicted KEEG pathways of sEV-associated miRs were carried out using DIANA TOOLS-mirPath v.3 as previously described [43].

### 4.7. Statistical Analysis

Differentially packaged miRs were then correlated with the available clinicopathological features of PCa patients. Overall survival (OS) and recurrence-free survival (RFS) were also analyzed using Kaplan–Meier as well as Cox biohazard regression analyses. Receiver operating characteristic (ROC) analyses were carried out to evaluate the diagnostic utility of these differentially packaged miRs collected from the plasma of PCa patients. A univariate Cox proportional hazards regression was performed to associate continuous sEV-associated miRs enrichment with OS and RFS. In addition, the Hazard Ratio (HR) was calculated for each sEV-associated miR as well as each clinicopathological parameter to evaluate their association with the OS and RFS. Significance of data was considered at *p*-value < 0.05 (log-rank test).

## 5. Conclusions

We report for the first time a set of sEV-associated miRs that can differentiate PCa from normal and stratify PCa patients according to their race and GS. The high levels of sEV-associated miR-1915-3p, miR-3692-3p and miR-5001-5p are associated with the improved overall survival of PCa patients. miR-1915-3p is the only classifier that is associated with longer RFS as an independent prognostic marker. Further studies are warranted to determine the role of these sEV-associated miRs in PCa progression and metastasis.

## Figures and Tables

**Table 1:** miR profiling of small extracellular vesicles (sEVs) collected from plasma of PCa patients compared to normal individuals, AA compared to CA PCa and high Gleason score compared to low Gleason score.

#	microRNA	FC	*p*-Value	FDR-Val	#	microRNA	FC	*p*-Value	FDR-Val
PCa Compared to Normal Subjects									
1	miR-4529-3p	354.7	0.0000	0.0001	44	miR-8060	2.6	0.0006	0.1526
2	**miR-3201**	30.6	0.0000	0.0013	45	miR-4644	2.6	0.0093	0.6487
3	miR-8084	28.5	0.0000	0.0001	46	let-7c-5p	2.6	0.0004	0.1242
4	miR-486-5p	21.5	0.0002	0.0801	47	miR-606	2.5	0.0002	0.0801
5	miR-26a-5p	21.4	0.0000	0.007	48	miR-4454	2.5	0.0181	0.8231
6	miR-92a-3p	14.5	0.0001	0.0502	49	HBII-85-6	2.5	0.0183	0.8272
7	miR-23a-3p	13.8	0.0008	0.1727	50	miR-330-3p	2.4	0.0024	0.3581
8	let-7b-5p	13.7	0.0174	0.8032	51	HBII-85-2	2.4	0.0003	0.0857
9	mir-7515	12.5	0.0000	0.0037	52	miR-6752-5p	2.4	0.0028	0.3737
10	miR-16-5p	12.3	0.0000	0.0014	53	miR-3910	2.4	0.017	0.7934
11	miR-6716-3p	12.0	0.0148	0.7721	54	miR-1275	2.4	0.0033	0.3971
12	miR-126-3p	10.9	0.0000	0.0111	55	miR-1288-5p	2.3	0.0226	0.8767
13	miR-320c	10.1	0.0001	0.0694	56	miR-4690-5p	2.3	0.0001	0.0502
14	miR-3128	9.7	0.0002	0.0801	57	miR-423-5p	2.3	0.0103	0.6708
15	miR-320a	8.2	0.0017	0.2835	58	miR-6780b-5p	2.3	0.0263	0.8767
16	miR-8075	7.5	0.0003	0.0937	59	miR-2392	2.2	0.0041	0.4217
17	miR-320b	7.2	0.0018	0.3023	60	miR-6801-5p	2.2	0.0206	0.8767
18	miR-23b-3p	6.8	0.0000	0.0108	61	miR-3074-3p	2.2	0.0345	0.8924
19	miR-103a-3p	6.1	0.0000	0.0067	62	miR-6807-5p	2.1	0.0024	0.3581
20	let-7a-5p	5.4	0.0001	0.0502	63	U3	2.1	0.0007	0.158
21	miR-619-5p	5.3	0.0000	0.0067	64	miR-19b-3p	2.1	0.0115	0.6907
22	miR-320d	5.2	0.0004	0.1242	65	mir-338	2.1	0.003	0.3928
23	mir-6798	5.2	0.0374	0.8924	66	miR-4423-3p	2.0	0.0055	0.514
24	SNORD59BL2	4.5	0.0464	0.9493	67	miR-548ac	2.0	0.0041	0.4217
25	miR-4445-3p	4.4	0.0033	0.3971	68	SNORA1	2.0	0.0307	0.8767
26	mir-520g	4.2	0.0002	0.0743	69	SNORA1B	2.0	0.0307	0.8767
27	mir-520h	4.2	0.0002	0.0743	70	miR-4739	−2.1	0.0128	0.7386
28	miR-107	4.1	0.0016	0.2835	71	mir-6722	−2.1	0.0285	0.8767
29	miR-6514-3p	3.8	0.0404	0.8933	72	miR-6511b-5p	−2.2	0.0413	0.8981
30	SNORD116-29L1	3.7	0.0146	0.7721	73	miR-1281	−2.4	0.0008	0.1623
31	mir-4275	3.6	0.0094	0.6487	74	miR-6511a-5p	−2.4	0.0463	0.9493
32	miR-150-5p	3.6	0.0014	0.2697	75	miR-4745-5p	−2.5	0.0136	0.7621
33	miR-24-3p	3.5	0.0058	0.5311	76	miR-6791-5p	−2.6	0.0336	0.8877
34	SNORD113-30	3.4	0.0391	0.8924	77	miR-6732-5p	−2.8	0.0479	0.9574
35	HBII-85-8	3.3	0.0055	0.514	78	miR-6800-5p	−3.2	0.0072	0.6192
36	mir-365a	3.2	0.0365	0.8924	79	miR-6789-5p	−3.8	0.0109	0.6765
37	mir-365a	3.2	0.0305	0.8767	80	miR-4516	−4.6	0.0144	0.7718
38	miR-342-3p	2.9	0.0015	0.2827	81	miR-6869-5p	−6.4	0.0304	0.8767
39	miR-335-5p	2.9	0.0051	0.5024	82	**miR-6068**	−8.2	0.0036	0.4123
40	SNORD116-21	2.7	0.0038	0.4123	83	miR-4487	−8.5	0.0213	0.8767
41	let-7d-5p	2.7	0.0063	0.5624	84	**miR-5001-5p**	−12.6	0.0026	0.3615
42	miR-1182	2.7	0.0214	0.8767	85	miR-4467	−25.5	0.0127	0.7374
43	miR-3151-5p	2.6	0.0087	0.6403	-	-	-	-	-
**AA compared to CA men with PCa**									
1	**miR-6716-5p**	3.41	0.0004	0.9126	11	miR-6729-5p	−2.13	0.0247	0.9527
2	miR-6510-5p	2.57	0.0469	0.9527	12	mir-6776	−2.14	0.0376	0.9527
3	miR-3175	2.42	0.0147	0.9527	13	miR-6782-5p	−2.21	0.0063	0.9527
4	miR-6743-5p	2.26	0.0341	0.9527	14	miR-1273g-3p	−2.27	0.0156	0.9527
5	miR-5004-5p	2.23	0.0314	0.9527	15	miR-99b-5p	−2.27	0.0431	0.9527
6	miR-4723-5p	2.21	0.0157	0.9527	16	miR-4725-3p	−2.34	0.0138	0.9527
7	miR-6076	2.08	0.0103	0.9527	17	**miR-1915-3p**	−2.57	0.0071	0.9527
8	**miR-3944-5p**	2.02	0.0029	0.9527	18	miR-6821-5p	−2.63	0.0322	0.9527
9	snoU13	−2.01	0.0072	0.9527	19	miR-6500-5p	−3.09	0.0131	0.9527
10	miR-3619-5p	−2.04	0.0091	0.9527	-	-	-	-	-
**High Gleason Score (HGS) compared to low GS (LGS) PCa patients**									
1	miR-6727-5p	5.2	0.0134	0.9643	23	miR-7975	2.2	0.0374	0.9643
2	miR-6125	4.1	0.0036	0.9643	24	**miR-3939**	2.2	0.0017	0.9643
3	miR-6869-5p	4.0	0.031	0.9643	25	miR-635	2.2	0.0086	0.9643
4	miR-3621	3.9	0.0261	0.9643	26	miR-3126-3p	2.2	0.0049	0.9643
5	miR-6858-5p	3.4	0.0262	0.9643	27	miR-770-5p	2.1	0.0044	0.9643
6	**miR-5189-5p**	3.2	0.0017	0.9643	28	snoU13	2.1	0.0247	0.9643
7	mir-4737	2.9	0.0023	0.9643	29	miR-140-3p	2.0	0.0418	0.9643
8	miR-5094	2.8	0.0011	0.9643	30	miR-4454	2.0	0.0161	0.9643
9	**miR-3692-3p**	2.8	0.0019	0.9643	31	mir-5092	2.0	0.0266	0.9643
10	snoU13	2.8	0.0188	0.9643	32	mir-3154	2.0	0.0397	0.9643
11	miR-4269	2.8	0.0189	0.9643	33	miR-758-5p	−2	0.0464	0.9643
12	miR-6741-5p	2.7	0.0089	0.9643	34	miR-4504	−2.1	0.0047	0.9643
13	miR-1469	2.7	0.013	0.9643	35	miR-6837-5p	−2.2	0.0387	0.9643
14	mir-4737	2.7	0.0032	0.9643	36	miR-107	−2.2	0.0036	0.9643
15	miR-574-3p	2.5	0.0025	0.9643	37	mir-658	−2.3	0.0009	0.9643
16	miR-6723-5p	2.5	0.0075	0.9643	38	miR-3174	−2.4	0.0478	0.9643
17	miR-196b-3p	2.5	0.0058	0.9643	39	miR-320a	−2.4	0.0053	0.9643
18	miR-378h	2.4	0.0018	0.9643	40	miR-320b	−3.1	0.0043	0.9643
19	miR-6786-5p	2.3	0.0203	0.9643	41	miR-642a-3p	−3.3	0.0134	0.9643
20	miR-3935	2.3	0.0339	0.9643	42	let-7b-5p	−5.0	0.0392	0.9643
21	miR-187-5p	2.3	0.0035	0.9643	43	miR-106b-5p	−5.8	0.0296	0.9643
22	miR-5703	2.3	0.003	0.9643	-	-	-	-	-

Bold: selected miRs to be validated by qPCR; FC: fold change 2.0; Val: value; FDR: false discovery rate; # sequence number.

**Table 2:** Differential expression of sEV-associated miRs in PCa patients compared to normal individuals in confirmatory cohorts.

sEV-Associated miRs	PCa (*N* = 150)	Normal (*N* = 30)	Trend	*p*-Value
	Mean ΔCT ± SEM	Mean ΔCT ± SEM		
miR-6068 (ΔCT)	−0.556 ± 0.125	−2.03 ± 0.138	Down	<0.001
miR-1915-3p (ΔCT)	−0.709 ± 0.09	0.5675 ± 0.085	Up	0.001
miR-3201 (ΔCT)	−16.38 ± 0.163	−16.60 ± 0.139	NS	0.315
miR-3692-3p (ΔCT)	1.368 ± 0.068	0.813 ± 0.103	Down	0.004
miR-3939 (ΔCT)	0.377 ± 0.124	−1.046 ± 0.151	Down	<0.001
miR-3944-5p (ΔCT)	−7.438 ± 0.119	−7.349 ± 0.107	NS	0.787
miR-5001-5p (ΔCT)	0.754 ± 0.101	0.684 ± 0.100	NS	0.623
miR-5189-5p (ΔCT)	−2.279 ± 0.099	−3.309 ± 0.197	Down	<0.001
miR-6716-5p (ΔCT)	−1.037 ± 0.076	−1.864 ± 0.119	Down	<0.001

**Table 3:** Diagnostic ability of individual and combined sEV-associated miRs (ΔCT) to differentiate PCa from normal individuals and AA from CA men in plasma collected from PCa patients in confirmatory cohorts.

Comparison	Predictor	miRs	Cutoff Value	Sensitivity	Specificity	AUC	95% CI		*p*-Value
							Lower	Upper	
PCa vs. Normal	Single miR	miR-5189-5p	−2.84	70%	65%	0.753	0.661	0.845	<0.001
		miR-3939	−0.975	84%	65%	0.809	0.736	0.881	<0.001
		miR-6068	−1.785	76%	70%	0.806	0.733	0.880	<0.001
		miR-1915-3p	0.505	70%	70%	0.713	0.637	0.788	0.002
		miR-3692-3p	0.985	71%	65%	0.726	0.638	0.815	0.001
		miR-6716-5p	−1.5400	74%	70%	0.795	0.710	0.879	<0.001
	Combined miRs	miR-1915-3p and miR-6716				0.883	0.830	0.937	<0.001
		miR-1915-3p, miR-3692-3p, miR-3939, miR-6068 and miR-6716-5p				0.904	0.853	0.954	<0.001
AA vs. CA (W)	Single miR	miR-5189-5p	−0.130	67%	64%	0.649	0.508	0.790	0.048
		miR-3939	−0.135	64%	70%	0.705	0.572	0.838	0.007
		miR-6716-5p	−0.740	71%	65%	0.697	0.539	0.820	0.021
	Combined miRs	miR-3939 and miR-6716				0.754	0.623	0.885	0.001
		miR-3939, miR-5189 and miR-6716				0.761	0.632	0.890	0.001
AA vs. CA (NIH)	Single miR	miR-3692-3p	−0.705	68%	62%	0.706	0.597	0.814	0.001
		miR-6716-5p	−0.8700	70%	56%	0.665	0.552	0.779	0.008
	Combined miRs	miR-3692-3p and miR-6716-5p				0.752	0.649	0.855	<0.001
GS>7 vs. GS<7 (W)	Single miR	miR-1915-3p	−0.0805	73%	50%	0.662	0.523	0.801	0.031
		miR-3692-3p	−0.5600	61%	61%	0.661	0.519	0.805	0.037
	Combined miRs	miR-1915-3p and miR-3692-3p				0.683	0.544	0.822	0.019
GS ≥ 7 vs. GS < 7 (NIH)	Single miR	miR-1915-3p	1.3900	70%	50%	0.700	0.585	0.814	0.003
		miR-3692-3p	−0.3050	66%	52%	0.661	0.482	0.740	0.092
		miR-5001-5p	0.4900	65%	67%	0.697	0.588	0.806	0.002
	Combined miRs	miR-1915-3p, miR-3692-3p and miR-5001-5p				0.818	0.725	0.911	<0.001

**Table 4:** Differential expression of miRs in PCa patients based on race and Gleason score. miRs expression was evaluated by qPCR analysis in confirmatory cohorts. Log2 FC of sEV-associated miRs was used to compare the miR expression in AA and CA by Student t-test. Up: upregulated miRs, NS: non-significant miR expression.

Expression of miRs According to the Race								
Washington Cohort					NIH Cohort			
miRs	AA	CA	Expression	*p*-Value	AA	CA	Expression	*p*-Value
	Log2FC ± SEM	Log2FC ± SEM			Log2FC ± SEM	Log2FC ± SEM		
miR-1915-3p	−0.069 ± 0.120	−0.292 ± 0.10	NS	0.168	1.527 ± 0.12	1.533 ± 0.13	NS	0.971
miR-3201	−0.228 ± 0.378	0.237 ± 0.32	NS	0.352	−0.031 ± 0.30	−0.699 ± 0.31	NS	0.123
miR-3692-3p	−0.476 ± 0.135	−0.577 ± 0.07	NS	0.516	−0.210 ± 0.14	−0.921 ± 0.14	**Up**	<0.001 *
miR-3939	0.225 ± 0.160	−0.421 ± 0.15	**Up**	0.005 *	−2.037 ± 0.16	−2.483 ± 0.21	NS	0.121
miR-3944-5p	0.581 ± 0.222	0.376 ± 0.22	NS	0.510	−0.074 ± 0.22	−0.266 ± 0.25	NS	0.562
miR-5001-5p	−0.960 ± 0.199	−1.055 ± 0.19	NS	0.726	0.724 ± 0.13	0.384 ± 0.15	NS	0.085
miR-5189-5p	0.075 ± 0.175	−0.051 ± 0.20	**Up**	0.033 *	−1.546 ± 0.12	−1.604 ± 0.17	NS	0.780
miR-6716-5p	−0.052 ± 0.146	−1.106 ± 0.15	**Up**	0.007 *	−0.543 ± 0.11	−1.100 ± 0.14	**Up**	0.003 *
**Expression of miRs according to Gleason score**								
**Washington cohort**					**NIH cohort**			
**miRs**	**GS**	**Log2FC ± SEM**		***p*-value**	**Log2FC ± SEM**		**ANOVA *p*-value**	
miR-1915-3p	<7	0.012 ± 0.13		0.015 *	1.219 ± 0.14		0.008 *	
	=7				1.792 ± 0.15			
	>7	−0.373 ± 0.0.08			1.683 ± 0.12			
miR-3201	<7	−0.081 ± 0.41		0.733	−0.303 ± 0.34		0.948	
	=7				−0.465 ± 0.44			
	>7	0.090 ± 0.28			−0.327 ± 0.35			
miR-3692-3p	<7	−0.344 ± 0.11		0.015 *	−0.266 ± 0.19		0.010 *	
	=7				−0.442 ± 0.16			
	>7	−0.710 ± 0.10			−0.993 ± 0.16			
miR-3939	<7	0.000 ± 0.20		0.361	−2.351 ± 0.20		0.783	
	=7				−2.147 ± 0.28			
	>7	−0.217 ± 0.13			−2.338 ± 0.21			
miR-3944-5p	<7	0.572 ± 0.21		0.549	−0.234 ± 0.26		0.918	
	=7				−0.075 ± 0.33			
	>7	0.385 ± 0.23			−0.200 ± 0.27			
miR-5001-5p	<7	−0.912 ± 0.20		0.485	0.236 ± 0.12		0.032 *	
	=7				0.866 ± 0.22			
	>7	−1.103 ± 0.19			0.561 ± 0.15			
miR-5189-5p	<7	−0.107 ± 0.20		0.437	−1.780 ± 0.17		0.253	
	=7				−1.362 ± 0.20			
	>7	−0.322 ± 0.19			−1.585 ± 0.16			
miR-6716-5p	<7	−0.712 ± 0.14		0.350	−1.041 ± 0.14		0.187	
	=7				−0.609 ± 0.19			
	>7	−0.923 ± 0.14			−0.844 ± 0.15			
miR-6068	<7	−1.48 ± 0.34		0.772	−2.64 ± 0.39		0.015 *	
	=7				−1.37 ± 0.32			
	>7	−1.63 ± 0.36			−1.38 ± 0.32			

* depicts significance at *p* < 0.05.

**Table 5:** Univariate and multivariate Cox regression analysis assessing the association of the sEV-associated miRs as well as PCa clinicopathological parameters with overall survival (OS). * refers to significance at *p*-value < 0.05.

Parameters	Categories	Univariate				Multivariate			
		HR	95.0% CI for HR		*p*-Value	HR	95.0% CI for HR		*p*-Value
			Lower	Upper			Lower	Upper	
Age	<mean vs. ≥mean	2.157	0.812	5.733	0.123	3.663	1.066	12.585	0.039 *
Race	AA vs. CA	1.373	0.549	3.434	0.498	3.169	1.082	9.278	0.035 *
Gleason Score	GS < 7 vs. ≥7	1.022	0.383	2.728	0.965	0.718	0.206	2.499	0.602
PSA	<median vs. ≥median	1.562	0.604	4.038	0.357	1.506	0.145	2.191	0.408
Stage	I, II vs. III, IV	0.659	0.191	2.279	0.51	0.564	0.601	2.935	0.483
Smoke Status	Smoker vs. non-smoker	1.166	0.658	2.065	0.512	1.328	0.547	4.147	0.428
miR-5189-3p	<mean Log2FC vs. ≥mean Log2FC	0.694	0.272	1.77	0.455	3.884	0.932	16.177	0.062
miR-1915-5p	<mean Log2FC vs. ≥mean Log2FC	0.264	0.103	0.682	0.006 *	0.217	0.053	0.877	0.033 *
miR-3692-3p	<mean Log2FC vs. ≥mean Log2FC	0.366	0.13	1.026	0.056	0.222	0.064	0.77	0.018 *
miR-5001-5p	<mean Log2FC vs. ≥mean Log2FC	0.399	0.155	1.025	0.056	0.609	0.151	2.457	0.486

**Table 6:** Univariate and multivariate Cox regression analysis assessing the association of the sEV-associated miRs as well as PCa clinicopathological parameters with recurrence-free survival (RFS). * refers to significance at *p*-value < 0.05.

Parameter	Categories	Univariate				Multivariate			
		HR	95% CI for HR		*p*-Value	HR	95% CI for HR		*p*-Value
			Lower	Upper			Lower	Upper	
Age	<mean vs. ≥mean	1.023	0.381	2.751	0.963	0.951	0.321	2.82	0.982
Race	AA vs. CA	1.617	0.586	4.46	0.354	2.136	0.793	6.172	0.161
Gleason Score	GS < 7 vs. ≥7	1.478	0.499	4.381	0.481	1.472	0.383	5.661	0.573
PSA	<median vs. ≥median	2.543	0.852	7.587	0.094	2.772	0.757	10.142	0.123
Stage	I, II vs. III, IV	1.533	0.48	4.892	0.47	1.031	0.288	3.69	0.693
Smoke Status	Smoker vs. non-smoker	1.422	0.763	2.652	0.268	1.242	0.624	2.471	0.536
miR-1915-5p (Log2FC)	<mean Log2FC vs. ≥mean Log2FC	0.286	0.099	0.828	0.021 *	0.288	0.067	0.788	0.018 *

## Data Availability

The generated data of the current study are available upon request.

## Supplementary Materials

The following Supplementary Materials are available online at https://www.mdpi.com/article/10.3390/cancers13205236/s1. Figure S1. Expression of surface markers proteins in small extracellular (sEVs) vesicles derived from plasma of PCa patients; Figure S2: Pathway prediction for sEV-associated miR-3201, miR-5001-5p, miR-6068, miR-6716-5p, miR-1915-3p, miR-3944-5p, miR-3939, miR-3692-3p and miR-5189-5p, Figure S3: Overall survival analysis for prostate cancer patients with different clinicopathological features. Kaplan–Meier analysis shows no association between the tested parameters and the overall survival of prostate cancer patients, Figure S4: Recurrence-free survival analysis for prostate cancer patients with different clinicopathological features. Table S1: List of blood samples collected from PCa and race-matched healthy individuals for conducting sEV-associated miR profiling, Table S2: Clinical information of PCa samples and their age- and race-matched healthy individuals (confirmatory cohorts), Table S3: qNano analysis for measuring the size and concentration of small extracellular vesicles (sEVs) derived from plasma of PCa patients, Table S4: miR profiling of small extracellular vesicles (sEVs) collected from plasma of PCa patients compared to normal individuals, AA compared to CA PCa and high Gleason score compared to low Gleason score when fold change at 1.5 was considered, Table S5: Diagnostic ability of individual and combined circulating small extracellular vesicle-associated miRs (Log2FC*) to segregate Gleason Scores of AA and CA PCa patients (Washington cohort), Table S6: Diagnostic ability of individual and combined circulating small extracellular vesicle-associated miRs (Log2FC*) to segregate Gleason Scores of AA and CA PCa patients (NIH cohort).
