# Supplementary material for: Small Extracellular Vesicle-Derived microRNAs Stratify Prostate Cancer Patients According to Gleason Score, Race and Associate with Survival of African American and Caucasian Men
Source: Cancers (Basel). 2021 Oct 19;13(20):5236. doi: 10.3390/cancers13205236 (PMC8533757; doi:10.3390/cancers13205236)

Supplementary Information

Small extracellular vesicles-derived microRNAs stratify prostate cancer patients according to Gleason score, race and associate with survival of African American and Caucasian men

Hamdy E.A. Ali[1], Mohamed S.A. Gaballah[1,2], Rofaida Gaballa[1], Shahenda Mahgoub[2], Zeinab A. Hassan[2], Eman Toraih[3], Bettina F. Drake[4], Zakaria Y. Abd Elmageed[1, 5#]

[1] Department of Pharmaceutical Sciences, Rangel College of Pharmacy, Texas A&M University, College Station, TX 77843, USA
[2] Department of Radiobiological Applications, Nuclear Research Center, Atomic Energy Authority, Cairo, Egypt
[3] Department of Biochemistry and Molecular Biology, Faculty of Pharmacy, Helwan University, Cairo 11795, Egypt
[4] Department of Surgery, Tulane University School of Medicine, 1430 Tulane Avenue, New Orleans, LA 70112, USA
[5] Division of Public Health Sciences, Washington University School of Medicine, Saint Louis, MO 63110, USA
[6] Department of Pharmacology, Edward Via College of Osteopathic Medicine, University of Louisiana at Monroe, Monroe, LA 71203, USA

Running Title: Small extracellular vesicle-associated miRs as diagnostic and prognostic markers in prostate cancer

#Correspondence to:

Zakaria Y. Abd Elmageed, PhD

Department of Pharmacology
Edward Via College of Osteopathic Medicine
University of Louisiana at Monroe
4408 Bon Aire Drive
Monroe, LA 71203
Phone: 318-342-7185
Email: zelmageed@ulm.vcom.edu

Table S1. List of blood samples collected from PCa and race-matched healthy individuals for conducting sEV-associated miR profiling

**Group**	**1. Age**		**Race**	**PSA**	**Stage**	**GS**
	Individual	Mean ± SD		Mean ±		
Group 1 (PCa)	45	53.2 0 ± 8.01	CA	9.4 ± 5.96	pT2c	6
	55				pT2c	6
	49				pT2c	6
	51				pT2c	6
	66				pT2c	6
	66				pT2c	6
Group 2 (PCa)	47	53.16 ± 5.53	AA	7.7 ± 3.96	pT2c	6
	48				pT2c	6
	52				pT2c	6
	54				pT3a	6
	56				pT2c	6
	62				pT2c	6
Group 3 (PCa)	61	65.66 ± 5.39	CA	15.1 ± 4.82	pT3a	8
	60				pT2c	8
	64				pT3a	9
	74				pT3a	9
	65				pT4	9
	70				pT2c	8
Group 4 (PCa)	60	64.50 ± 5.99	AA	14.7 ± 6.14	pT3a	8
	61				pT2c	8
	73				pT2c	9
	59				pT2c	9
	71				pT2c	8
	63				pT3a	9
Group 5 (Normal)	58	56.67 ± 5.13	CA			
	54					
	62					
	63					
	51					
	52					
Gr ou p 6	57	56.83 ± 3.13	AA			
	53					

	59					
	59					
	53					
	60					

AA: African American; CA: Caucasian American; GS: Gleason score; SD: standard deviation

Table S2. Clinical information of PCa samples and their age and race-matched healthy individuals (confirmatory cohorts)

Variable	Washington Cohort				NIH Cohort	
	Normal		PCa		PCa	
	AA	CA	AA	CA	AA	CA
**Total, n**	15	15	30	30	45	45
**Age, mean ± SD (Y)**	56 ± 5.3	57 ± 3.4	61 ± 9.2	62 ± 8.8	64 ± 7.08	64 ± 6.96
**PSA, mean ± SD (ng/ml)**			6.9 ± 3.4	8.6 ± 6.7	13.4 ± 16.5	12.3 ± 22.3
**Gleason score (n)**						
**GS <7**			15	15	15	15
**GS=7**			0	0	15	15
**GS>7**			15	15	15	15
**Clinical stage (n)**						
**T1c**			26	20	5	6
**T2a**			3	6	9	7
**T2b**			0	0	21	20
**T2c**			0	1	0	0
**T3a**			0	0	6	9
**T3b**			0	0	0	0
**T4**			0	0	4	3
**Smoke Status**						
**Never**			10	15	14	17
**Current**			7	0	16	10
**Former**			12	13	15	18

AA: African American; CA: Caucasian American

Table S3. qNano analysis for measuring the size and concentration of small extracellular vesicles (sEVs) derived from plasma of PCa patients.

		**Particle diameter (nm)**			**Concentration (particles/mL)**	
Samples	Experiment	Mean	SD	Mode	Measured	Final Concentration
AA-LGS	Repeat-1	154	63.1	110	8.21E+10	1.64E+11
	Repeat-2	151	60	95	6.72E+10	1.34E+11
	Average	152.5	61.6	102.5	7.47E+10	1.49E+11
AA-HGS	Repeat-1	161	70.4	100	1.25E+11	2.51E+11
	Repeat-2	156	68.6	122	1.13E+11	2.26E+11
	Average	158.5	69.5	111.0	1.19E+11	2.385E+11
CA-LGS	Repeat-1	162	65.5	96	1.30E+11	2.59E+11
	Repeat-2	151	61.4	98	8.38E+10	1.68E+11
	Average	156.5	63.5	97	1.069E+11	2.135E+11
CA-HGS	Repeat-1	145	55.2	128	6.87E+10	1.37E+11
	Repeat-2	151	57.8	110	4.92E+10	9.84E+10
	Average	148.0	56.5	119	5.90E+10	1.177E+11

AA-HGS: AA men with high Gleason score; CA-HGS: CA men with high Gleason score
AA-LGS: AA men with low Gleason score; CA-LGS: CA men with low Gleason score

Table S4. miR profiling of small extracellular vesicles (sEVs) collected from plasma of PCa patients compared to normal individuals, AA compared to CA PCa and high Gleason score compared to low Gleason score when fold change at 1.5 was considered.

#	sEVs-miR	FC	*p*-value	FDR-	#	sEVs-miR	FC	*p*-	FDR-
**A. PCa compared to normal subjects**									
1	miR-4529-3p	354.7	0.0000	0.0001	94	ENSG00000202	1.8	0.016	0.7926
2	miR-3201	30.6	0.0000	0.0013	95	miR-489-3p	1.8	0.047	0.9574
3	miR-8084	28.5	0.0000	0.0001	96	miR-575	1.8	0.003	0.3971
4	miR-486-5p	21.5	0.0002	0.0801	97	ENSG00000238	1.8	0.032	0.8806
5	miR-26a-5p	21.4	0.0000	0.007	98	mir-4775	1.7	0.003	0.4123
6	miR-92a-3p	14.5	0.0001	0.0502	99	miR-6746-5p	1.7	0.010	0.6765
7	miR-23a-3p	13.8	0.0008	0.1727	100	mir-548g	1.7	0.015	0.7721
8	let-7b-5p	13.7	0.0174	0.8032	101	ENSG00000253	1.7	0.000	0.1526
9	mir-7515	12.5	0.0000	0.0037	102	ENSG00000212	1.7	0.018	0.8322
10	miR-16-5p	12.3	0.0000	0.0014	103	miR-6877-5p	1.7	0.023	0.8767
11	miR-6716-3p	12.0	0.0148	0.7721	104	miR-509-3-5p	1.7	0.036	0.8924
12	miR-126-3p	10.9	0.0000	0.0111	105	miR-548x-3p	1.7	0.004	0.4217
13	miR-320c	10.1	0.0001	0.0694	106	ENSG00000252	1.7	0.021	0.8767
14	miR-3128	9.7	0.0002	0.0801	107	miR-378a-3p	1.7	0.001	0.2835
15	miR-320a	8.2	0.0017	0.2835	108	mir-181b-2	1.7	0.002	0.3737
16	miR-8075	7.5	0.0003	0.0937	109	miR-27a-3p	1.7	0.025	0.8767
17	miR-320b	7.2	0.0018	0.3023	110	ENSG00000238	1.7	0.002	0.3615
18	miR-23b-3p	6.8	0.0000	0.0108	111	U83B	1.7	0.009	0.6487
19	miR-103a-3p	6.1	0.0000	0.0067	112	miR-151a-5p	1.7	0.015	0.7747
20	let-7a-5p	5.4	0.0001	0.0502	113	mir-3910-1	1.7	0.018	0.8312
21	miR-619-5p	5.3	0.0000	0.0067	114	mir-6816	1.7	0.019	0.8322
22	miR-320d	5.2	0.0004	0.1242	115	miR-4291	1.7	0.034	0.8924
23	mir-6798	5.2	0.0374	0.8924	116	miR-4804-5p	1.7	0.042	0.9059
24	ENSG00000252	4.5	0.0464	0.9493	117	miR-4478	1.7	0.013	0.7638
25	miR-4445-3p	4.4	0.0033	0.3971	118	mir-24-1	1.7	0.014	0.7721
26	mir-520g	4.2	0.0002	0.0743	119	miR-93-5p	1.7	0.035	0.8924
27	mir-520h	4.2	0.0002	0.0743	120	U57	1.7	0.014	0.7718
28	miR-107	4.1	0.0016	0.2835	121	ENSG00000252	1.7	0.026	0.8767
29	miR-6514-3p	3.8	0.0404	0.8933	122	ACA4	1.7	0.032	0.8806
30	ENSG00000252	3.7	0.0146	0.7721	123	ENSG00000207	1.6	0.005	0.514
31	mir-4275	3.6	0.0094	0.6487	124	ENSG00000238	1.6	0.002	0.3615
32	miR-150-5p	3.6	0.0014	0.2697	125	miR-200b-5p	1.6	0.008	0.6403
33	miR-24-3p	3.5	0.0058	0.5311	126	miR-4671-5p	1.6	0.020	0.8767
34	ENSG00000252	3.4	0.0391	0.8924	127	hsa-miR-4784	1.6	0.024	0.8767
35	HBII-85-8	3.3	0.0055	0.514	128	miR-197-3p	1.6	0.008	0.6361
36	mir-365a	3.2	0.0365	0.8924	129	ENSG00000238	1.6	0.013	0.7564
37	mir-365a	3.2	0.0305	0.8767	130	miR-4779	1.6	0.019	0.8424
38	miR-342-3p	2.9	0.0015	0.2827	131	ENSG00000253	1.6	0.023	0.8767

39	miR-335-5p	2.9	0.0051	0.5024	132	HBII-289	1.6	0.003	0.4123
40	ENSG00000252	2.7	0.0038	0.4123	133	mir-4466	1.6	0.027	0.8767
41	let-7d-5p	2.7	0.0063	0.5624	134	miR-4258	1.6	0.002	0.3551
42	miR-1182	2.7	0.0214	0.8767	135	miR-5681a	1.6	0.010	0.6669
43	miR-3151-5p	2.6	0.0087	0.6403	136	mir-532	1.6	0.029	0.8767
44	miR-8060	2.6	0.0006	0.1526	137	ENSG00000252	1.6	0.005	0.5024
45	miR-4644	2.6	0.0093	0.6487	138	mir-620	1.6	0.035	0.8924
46	let-7c-5p	2.6	0.0004	0.1242	139	miR-6735-5p	1.6	0.043	0.9062
47	miR-606	2.5	0.0002	0.0801	140	miR-4704-5p	1.6	0.003	0.4164
48	miR-4454	2.5	0.0181	0.8231	141	miR-5695	1.6	0.029	0.8767
49	HBII-85-6	2.5	0.0183	0.8272	142	spike_in-	1.6	0.009	0.6622
50	miR-330-3p	2.4	0.0024	0.3581	143	gi:555853	1.6	0.026	0.8767
51	HBII-85-2	2.4	0.0003	0.0857	144	ENSG00000251	1.5	0.006	0.5666
52	miR-6752-5p	2.4	0.0028	0.3737	145	ENSG00000265	1.5	0.006	0.5666
53	miR-3910	2.4	0.0170	0.7934	146	ENSG00000201	1.5	0.024	0.8767
54	miR-1275	2.4	0.0033	0.3971	147	miR-222-5p	1.5	0.027	0.8767
55	miR-1288-5p	2.3	0.0226	0.8767	148	miR-3200-3p	1.5	0.034	0.8924
56	miR-4690-5p	2.3	0.0001	0.0502	149	mir-3127	1.5	0.002	0.3902
57	miR-423-5p	2.3	0.0103	0.6708	150	miR-922	1.5	0.005	0.5024
58	miR-6780b-5p	2.3	0.0263	0.8767	151	miR-4797-5p	1.5	0.003	0.3971
59	miR-2392	2.2	0.0041	0.4217	152	ENSG00000201	1.5	0.009	0.6487
60	miR-6801-5p	2.2	0.0206	0.8767	153	miR-718	1.5	0.009	0.6487
61	miR-3074-3p	2.2	0.0345	0.8924	154	ENSG00000238	1.5	0.032	0.8806
62	miR-6807-5p	2.1	0.0024	0.3581	155	ENSG00000238	-1.5	0.032	0.8806
63	ENSG00000252	2.1	0.0007	0.158	156	14qII-20	-1.5	0.008	0.6361
64	miR-19b-3p	2.1	0.0115	0.6907	157	mir-1208	-1.5	0.036	0.8924
65	mir-338	2.1	0.0030	0.3928	158	ENSG00000212	-1.6	0.031	0.8806
66	miR-4423-3p	2.0	0.0055	0.514	159	miR-4492	-1.6	0.010	0.6765
67	miR-548ac	2.0	0.0041	0.4217	160	miR-1178-5p	-1.6	0.032	0.8806
68	ACA1	2.0	0.0307	0.8767	161	ENSG00000200	-1.6	0.024	0.8767
69	ENSG00000199	2.0	0.0307	0.8767	162	miR-6075	-1.6	0.023	0.8767
70	mir-3128	2.0	0.0002	0.0801	163	mir-1181	-1.7	0.029	0.8767
71	mir-4753	2.0	0.0108	0.6765	164	mir-6858	-1.7	0.010	0.6669
72	HBII-85-26	2.0	0.0405	0.8933	165	miR-211-3p	-1.9	0.027	0.8767
73	miR-6820-5p	2.0	0.0403	0.8933	166	mir-550a-1	-1.9	0.047	0.953
74	mir-8075	2.0	0.0355	0.8924	167	mir-550a-2	-1.9	0.047	0.953
75	miR-204-3p	2.0	0.0365	0.8924	168	mir-550a-3	-1.9	0.047	0.953
76	miR-8054	1.9	0.0006	0.1558	169	ENSG00000251	-1.9	0.008	0.6403
77	ENSG00000238	1.9	0.0010	0.1941	170	miR-4739	-2.1	0.012	0.7386
78	mir-1260a	1.9	0.0252	0.8767	171	mir-6722	-2.1	0.028	0.8767
79	ENSG00000202	1.9	0.0007	0.158	172	miR-6511b-5p	-2.2	0.041	0.8981
80	let-7i-5p	1.9	0.0076	0.6266	173	miR-1281	-2.4	0.000	0.1623
81	mir-640	1.9	0.0232	0.8767	174	miR-6511a-5p	-2.4	0.046	0.9493
82	miR-3622a-5p	1.9	0.0235	0.8767	175	miR-4745-5p	-2.5	0.013	0.7621

83	miR-4684-3p	1.8	0.0238	0.8767	176	miR-6791-5p	-2.6	0.033	0.8877
84	mir-4679-1	1.8	0.0011	0.2062	177	miR-6732-5p	-2.8	0.047	0.9574
85	miR-3177-3p	1.8	0.0495	0.9675	178	miR-6800-5p	-3.2	0.007	0.6192
86	mir-4512	1.8	0.0095	0.6487	179	miR-6789-5p	-3.8	0.010	0.6765
87	U26	1.8	0.0285	0.8767	180	miR-4516	-4.6	0.014	0.7718
88	ENSG00000252	1.8	0.0034	0.3971	181	miR-6869-5p	-6.4	0.030	0.8767
89	ENSG00000252	1.8	0.0022	0.3551	182	**miR-6068**	-8.2	0.003	0.4123
90	ENSG00000253	1.8	0.0236	0.8767	183	miR-4487	-8.5	0.021	0.8767
91	miR-25-3p	1.8	0.0008	0.1623	184	**miR-5001-5p**	-12.6	0.002	0.3615
92	mir-6765	1.8	0.0073	0.6223	185	miR-4467	-25.5	0.012	0.7374
93	mir-6765	1.8	0.0073	0.6223					
**B. AA compared to CA men with PCa**									
1	**miR-6716-5p**	3.4	0.0004	0.9126	52	U71d	1.5	0.020	0.9527
2	miR-6510-5p	2.6	0.0469	0.9527	53	miR-93	1.5	0.034	0.9527
3	miR-3175	2.4	0.0147	0.9527	54	miR-378e	1.5	0.047	0.9527
4	miR-6743-5p	2.3	0.0341	0.9527	55	U49B	1.5	0.018	0.9527
5	miR-5004-5p	2.2	0.0314	0.9527	56	miR-152-5p	-1.5	0.012	0.9527
6	miR-4723-5p	2.2	0.0157	0.9527	57	ENSG00000252	-1.5	0.036	0.9527
7	miR-6076	2.1	0.0103	0.9527	58	miR-185	-1.5	0.038	0.9527
8	miR-3944-5p	2.0	0.0029	0.9527	59	miR-1343-3p	-1.5	0.039	0.9527
9	miR-6797-5p	2.0	0.0004	0.9126	60	miR-5588-3p	-1.5	0.043	0.9527
10	miR-524-5p	1.9	0.0209	0.9527	61	miR-218-1-3p	-1.5	0.001	0.9527
11	ENSG00000202	1.9	0.0064	0.9527	62	miR-1291	-1.5	0.013	0.9527
12	miR-4505	1.9	0.0005	0.9126	63	miR-4666a-5p	-1.5	0.014	0.9527
13	miR-520f	1.9	0.0006	0.9126	64	ENSG00000268	-1.5	0.018	0.9527
14	miR-4520b-3p	1.9	0.0208	0.9527	65	U70	-1.5	0.018	0.9527
15	miR-1226-5p	1.9	0.0209	0.9527	66	miR-154-5p	-1.5	0.001	0.9527
16	ENSG00000238	1.9	0.0222	0.9527	67	miR-26a-1	-1.5	0.018	0.9527
17	miR-4486	1.8	0.0164	0.9527	68	miR-636	-1.5	0.038	0.9527
18	miR-7856-5p	1.8	0.0266	0.9527	69	miR-3652	-1.6	0.022	0.9527
19	miR-483-5p	1.8	0.0097	0.9527	70	SNORA38B	-1.6	0.022	0.9527
20	miR-4446	1.8	0.007	0.9527	71	ENSG00000238	-1.6	0.022	0.9527
21	ENSG00000253	1.7	0.0008	0.9527	72	ENSG00000238	-1.6	0.020	0.9527
22	miR-4768-3p	1.7	0.0228	0.9527	73	miR-218-1	-1.6	0.021	0.9527
23	miR-4436a	1.7	0.0202	0.9527	74	miR-340-3p	-1.6	0.036	0.9527
24	miR-1183	1.7	0.0328	0.9527	75	miR-8078	-1.6	0.032	0.9527
25	ENSG00000252	1.7	0.0045	0.9527	76	miR-6833-3p	-1.6	0.017	0.9527
26	ENSG00000263	1.7	0.0045	0.9527	77	ENSG00000238	-1.6	0.025	0.9527
27	ENSG00000263	1.7	0.0045	0.9527	78	HBII-85-26	-1.7	0.011	0.9527
28	ENSG00000263	1.7	0.0045	0.9527	79	miR-7515	-1.7	0.027	0.9527
29	ENSG00000265	1.7	0.0045	0.9527	80	miR-631	-1.7	0.048	0.9527
30	ENSG00000265	1.7	0.0045	0.9527	81	miR-5696	-1.7	0.012	0.9527
31	ENSG00000265	1.7	0.0045	0.9527	82	miR-6756-5p	-1.7	0.049	0.9527
32	ENSG00000265	1.7	0.0045	0.9527	83	miR-3921	-1.7	0.013	0.9527

33	miR-5088-5p	1.7	0.0212	0.9527	84	miR-191-5p	-1.8	0.037	0.9527
34	miR-1468-3p	1.7	0.0115	0.9527	85	miR-141-5p	-1.8	0.012	0.9527
35	ENSG00000252	1.7	0.0396	0.9527	86	miR-638	-1.8	0.036	0.9527
36	miR-4257	1.6	0.0221	0.9527	87	miR-5680	-1.8	0.016	0.9527
37	miR-4711-5p	1.6	0.016	0.9527	88	ENSG00000238	-1.9	0.023	0.9527
38	miR-6865-5p	1.6	0.0445	0.9527	89	miR-548ae-1	-1.9	0.005	0.9527
39	ENSG00000200	1.6	0.0068	0.9527	90	hsa-let-7d-3p	-1.9	0.037	0.9527
40	miR-3153	1.6	0.0234	0.9527	91	ENSG00000239	-2.0	0.007	0.9527
41	miR-7977	1.6	0.0063	0.9527	92	miR-3619-5p	-2.0	0.009	0.9527
42	miR-6880-5p	1.6	0.0038	0.9527	93	miR-6729-5p	-2.1	0.024	0.9527
43	miR-4300	1.6	0.0116	0.9527	94	miR-6776	-2.1	0.037	0.9527
44	miR-4255	1.6	0.0163	0.9527	95	miR-6782-5p	-2.2	0.006	0.9527
45	HBII-52-32	1.6	0.0372	0.9527	96	miR-1273g-3p	-2.3	0.015	0.9527
46	ENSG00000238	1.5	0.0086	0.9527	97	miR-99b-5p	-2.3	0.043	0.9527
47	ENSG00000206	1.5	0.017	0.9527	98	miR-4725-3p	-2.3	0.013	0.9527
48	miR-382-3p	1.5	0.0476	0.9527	99	**miR-1915-3p**	-2.6	0.007	0.9527
49	miR-5696	1.5	0.0069	0.9527	100	miR-6821-5p	-2.6	0.032	0.9527
55	ENSG00000199	1.5	0.0092	0.9527	101	miR-6500-5p	-3.1	0.013	0.9527
51	miR-551b-5p	1.5	0.0152	0.9527					
**C. HGS compared to LGS PCa patients**									
1	miR-6727-5p	5.17	0.0134	0.9643	90	miR-4263	1.62	0.029	0.9643
2	miR-6125	4.09	0.0036	0.9643	91	miR-15a-3p	1.62	0.043	0.9643
3	miR-6869-5p	3.96	0.031	0.9643	92	miR-4521	1.61	0.014	0.9643
4	miR-3621	3.94	0.0261	0.9643	93	miR-7150	1.61	0.016	0.9643
5	miR-6858-5p	3.39	0.0262	0.9643	94	miR-4686	1.6	0.013	0.9643
6	miR-5189-5p	3.2	0.0017	0.9643	95	miR-150	1.6	0.015	0.9643
7	miR-4737	2.86	0.0023	0.9643	96	ENSG00000238	1.6	0.024	0.9643
8	miR-5094	2.84	0.0011	0.9643	97	miR-6862-5p	1.6	0.037	0.9643
9	**miR-3692-3p**	2.82	0.0019	0.9643	98	miR-6811	1.6	0.039	0.9643
10	ENSG00000238	2.82	0.0188	0.9643	99	miR-221	1.6	0.045	0.9643
11	miR-4269	2.79	0.0189	0.9643	100	ENSG00000252	1.6	0.047	0.9643
12	miR-6741-5p	2.71	0.0089	0.9643	101	miR-3972	1.59	0.014	0.9643
13	miR-1469	2.71	0.013	0.9643	102	ENSG00000238	1.59	0.046	0.9643
14	miR-4737	2.66	0.0032	0.9643	103	miR-6837-3p	1.57	0.017	0.9643
15	miR-574-3p	2.49	0.0025	0.9643	104	miR-4796-5p	1.57	0.020	0.9643
16	miR-6723-5p	2.47	0.0075	0.9643	105	14qI-4	1.56	0.013	0.9643
17	miR-196b-3p	2.45	0.0058	0.9643	106	miR-5195-5p	1.56	0.014	0.9643
18	miR-378h	2.42	0.0018	0.9643	107	ENSG00000212	1.56	0.017	0.9643
19	miR-6786-5p	2.3	0.0203	0.9643	108	miR-1273h-3p	1.56	0.019	0.9643
20	miR-3935	2.3	0.0339	0.9643	109	ENSG00000206	1.56	0.025	0.9643
21	miR-187-5p	2.27	0.0035	0.9643	110	miR-6820-3p	1.56	0.047	0.9643
22	miR-5703	2.26	0.003	0.9643	111	miR-363	1.55	0.046	0.9643
23	miR-7975	2.23	0.0374	0.9643	112	miR-548aq	1.55	0.048	0.9643
24	**miR-3939**	2.22	0.0017	0.9643	113	miR-1247-3p	1.54	0.018	0.9643

25	miR-635	2.2	0.0086	0.9643	114	ENSG00000238	1.54	0.021	0.9643
26	miR-3126-3p	2.19	0.0049	0.9643	115	miR-4776-5p	1.54	0.037	0.9643
27	miR-770-5p	2.12	0.0044	0.9643	116	miR-3160-2	1.53	0.018	0.9643
28	ENSG00000239	2.06	0.0247	0.9643	117	miR-3150b-5p	1.53	0.045	0.9643
29	miR-140-3p	2.04	0.0418	0.9643	118	miR-6765-3p	1.52	0.027	0.9643
30	miR-4454	2.01	0.0161	0.9643	119	miR-219b	1.5	0.035	0.9643
31	miR-5092	2.01	0.0266	0.9643	120	miR-4304	-1.5	0.044	0.9643
32	miR-3154	2.01	0.0397	0.9643	121	miR-5087	-1.51	0.020	0.9643
33	miR-6828	1.99	0.002	0.9643	122	HBII-52-34	-1.51	0.035	0.9643
34	miR-5704	1.99	0.0062	0.9643	123	miR-376b-3p	-1.51	0.043	0.9643
35	miR-3648	1.98	0.0058	0.9643	124	HBI-115	-1.52	0.022	0.9643
36	miR-8089	1.98	0.0067	0.9643	125	ENSG00000238	-1.52	0.023	0.9643
37	miR-92b-5p	1.97	0.021	0.9643	126	miR-888	-1.52	0.026	0.9643
38	miR-8074	1.95	0.0026	0.9643	127	miR-3674	-1.52	0.032	0.9643
39	ENSG00000238	1.94	0.011	0.9643	128	ENSG00000200	-1.52	0.040	0.9643
40	miR-2467-3p	1.91	0.0486	0.9643	129	ENSG00000252	-1.52	0.046	0.9643
41	miR-6078	1.9	0.0045	0.9643	130	miR-5682	-1.53	0.02	0.9643
42	miR-4718	1.89	0.0271	0.9643	131	miR-2116-5p	-1.53	0.020	0.9643
43	ENSG00000252	1.88	0.0198	0.9643	132	miR-556-5p	-1.53	0.022	0.9643
44	ENSG00000262	1.88	0.0198	0.9643	133	miR-374c	-1.53	0.028	0.9643
45	ENSG00000238	1.88	0.0361	0.9643	134	miR-7973-2	-1.53	0.040	0.9643
46	ENSG00000264	1.87	0.004	0.9643	135	miR-8077	-1.54	0.024	0.9643
47	ENSG00000264	1.85	0.0034	0.9643	136	ENSG00000238	-1.54	0.028	0.9643
48	miR-2277-5p	1.85	0.0046	0.9643	137	miR-6740-5p	-1.54	0.044	0.9643
49	miR-188	1.85	0.0334	0.9643	138	miR-5708	-1.55	0.016	0.9643
50	miR-4740	1.84	0.0378	0.9643	139	miR-4765	-1.55	0.032	0.9643
51	miR-6879-5p	1.82	0.0177	0.9643	140	miR-544b	-1.56	0.013	0.9643
52	miR-937-3p	1.81	0.0139	0.9643	141	ENSG00000252	-1.56	0.013	0.9643
53	miR-1913	1.8	0.0048	0.9643	142	miR-4327	-1.56	0.020	0.9643
54	miR-6072	1.78	0.0043	0.9643	143	ENSG00000252	-1.57	0.012	0.9643
55	ENSG00000239	1.78	0.0079	0.9643	144	miR-4669	-1.57	0.025	0.9643
56	miR-6876-5p	1.78	0.0133	0.9643	145	miR-3663-3p	-1.57	0.033	0.9643
57	miR-1229-3p	1.78	0.03	0.9643	146	miR-6776-5p	-1.58	0.013	0.9643
58	miR-8072	1.77	0.0336	0.9643	147	miR-3153	-1.58	0.016	0.9643
59	ENSG00000212	1.77	0.044	0.9643	148	ENSG00000238	-1.59	0.013	0.9643
60	miR-3150b	1.77	0.0467	0.9643	149	miR-516a-1	-1.59	0.046	0.9643
61	miR-6851	1.76	0.009	0.9643	150	miR-516a-2	-1.59	0.046	0.9643
62	ENSG00000238	1.75	0.0105	0.9643	151	miR-3155b	-1.61	0.011	0.9643
63	miR-187	1.75	0.0109	0.9643	152	miR-1178-5p	-1.61	0.023	0.9643
64	miR-3687	1.75	0.0183	0.9643	153	miR-20a-5p	-1.65	0.049	0.9643
65	miR-4786	1.73	0.0109	0.9643	154	HBII-52-33	-1.66	0.014	0.9643
66	miR-489-3p	1.73	0.0313	0.9643	155	miR-3925	-1.67	0.040	0.9643
67	miR-6850-3p	1.72	0.0198	0.9643	156	miR-4731-3p	-1.69	0.013	0.9643
68	miR-941-1	1.72	0.0352	0.9643	157	miR-342-3p	-1.73	0.041	0.9643

69	miR-941-2	1.72	0.0352	0.9643	158	miR-142-5p	-1.76	0.025	0.9643
70	miR-941-3	1.72	0.0352	0.9643	159	miR-204-3p	-1.76	0.041	0.9643
71	miR-941-4	1.72	0.0352	0.9643	160	miR-4471	-1.77	0.034	0.9643
72	miR-4706	1.71	0.0255	0.9643	161	mgU12-22-U4-8	-1.78	0.024	0.9643
73	miR-504-3p	1.71	0.0482	0.9643	162	miR-6769b-5p	-1.8	0.021	0.9643
74	miR-4442	1.7	0.0128	0.9643	163	miR-4293	-1.84	0.029	0.9643
75	miR-4753	1.7	0.0351	0.9643	164	miR-3160-3p	-1.84	0.031	0.9643
76	miR-3155a	1.67	0.0128	0.9643	165	gi:555853	-1.85	0.029	0.9643
77	miR-4279	1.67	0.0168	0.9643	166	miR-370-3p	-1.89	0.012	0.9643
78	ENSG00000207	1.67	0.0275	0.9643	167	miR-1182	-1.98	0.022	0.9643
79	miR-147b	1.65	0.0197	0.9643	168	miR-758-5p	-2.04	0.046	0.9643
80	miR-4645	1.65	0.0242	0.9643	169	miR-4504	-2.05	0.004	0.9643
81	miR-4507	1.65	0.0354	0.9643	170	miR-6837-5p	-2.15	0.038	0.9643
82	miR-193b-5p	1.64	0.0249	0.9643	171	miR-107	-2.17	0.003	0.9643
83	miR-6805	1.63	0.0089	0.9643	172	miR-658	-2.28	0.000	0.9643
84	miR-153-2	1.63	0.0282	0.9643	173	miR-3174	-2.38	0.047	0.9643
85	miR-4638-3p	1.63	0.0386	0.9643	174	miR-320a	-2.44	0.005	0.9643
86	miR-411-5p	1.63	0.0437	0.9643	175	miR-320b	-3.1	0.004	0.9643
87	miR-7-2-3p	1.62	0.0131	0.9643	176	miR-642a-3p	-3.33	0.013	0.9643
88	miR-2114	1.62	0.0186	0.9643	177	hsa-let-7b-5p	-5	0.039	0.9643
89	miR-4417	1.62	0.0194	0.9643	178	miR-106b-5p	-5.79	0.029	0.9643

Bold miR: selected sEV-associated miRs to be validated by qPCR; FC: fold change at 1.5; Val: value; FDR: false discovery rate

Table S5. Diagnostic ability of individual and combined circulating small extracellular vesicle-associated miRs (Log2FC*) to segregate Gleason Scores of AA and CA PCa patients (Washington cohort).

Groups	Predictor	miR	Cut-off value	Sensitivity	Specificity	AUC	95% CI		*p*-value
							Lower	Upper	
AAHGS vs CAHGS	Single miR	miR-3692-3p	-0.680	71%	64%	0.719	0.526	0.913	0.048
AAHGS vs CALGS	Single miR	miR-3692-3p (ΔCT)*	1.475	79%	69%	0.776	0.584	0.967	0.013
AALGS vs CAHGS	Single miR	miR-5189-5p	-0.025	73%	67%	0.709	0.520	0.898	0.051
		miR-3939	-0.280	79%	60%	0.752	0.576	0.926	0.021
		miR-1915-3p	-0.140	73%	60%	0.782	0.619	0.946	0.008
		miR-3692-3p	-0.745	71%	50%	0.702	0.506	0.897	0.070
		miR-6716-5p	-0.740	87%	50%	0.712	0.523	0.900	0.052
	Combined miRs	miR-1915-3p & miR-6716-5p				0.843	0.692	0.993	0.002
		miR-1915-3p & miR-3939				0.814	0.657	0.971	0.004
		miR-1915-3p, miR-3939 & miR-6716-5p				0.883	0.751	1.000	0.001
AALGS vs CALGS	Single miR	miR-5189-5p	-0.855	87%	60%	0.720	0.520	0.912	0.040
		miR-3939	-0.290	79%	67%	0.812	0.658	0.966	0.004
		miR-1915-3p	-0.900	80%	67%	0.776	0.605	0.946	0.010
		miR-3692-3p	-0.525	79%	57%	0.719	0.526	0.913	0.048
		miR-6716-5p	-0.785	87%	79%	0.795	0.620	0.970	0.007
	Combined miRs	miR-3939 & miR-3692-3p				0.863	0.728	0.997	0.001
		miR-3692-3p & miR-6716-5p				0.824	0.665	0.984	0.004
		miR-1915-3p, miR-3692 & miR-6716-5p				0.888	0.762	1.000	0.001
AAHGS vs AALGS	Single miR	miR-3939	0.695	92%	50%	0.730	0.536	0.923	0.039
		miR-1915-3p	-0.295	73%	87%	0.847	0.709	0.984	0.001
		miR-3692-3p	-0.640	71%	86%	0.860	0.726	0.995	0.001
	Combined miRs	miR-1915-3p & miR-3692-3p				0.872	0.746	0.999	0.001

AAHGS: AA men with high Gleason score; CAHGS: Caucasian men with high Gleason score; AALGS: AA men with low Gleason score; CALGS: CA men with low Gleason score

Table S6. Diagnostic ability of individual and combined circulating small extracellular vesicle-associated miRs (Log2FC*) to segregate Gleason Scores of AA and CA PCa patients (NIH cohort).

Groups	Predictor	microRNA	Cut-off	Sensitivity	Specificity	AUC	95% CI		p-value
							Lower	Upper	
AAHGS vs AALGS	Single miR	miR-1915-3p	0.875	0.930	0.500	0.773	0.600	0.946	0.041
		miR-3692-	-0.325	0.790	0.770	0.764	0.581	0.948	0.015
		miR-5001-	0.400	0.730	0.600	0.744	0.565	0.924	0.023
		miR-6716-	-0.635	0.600	0.850	0.692	0.490	0.894	0.089
	Combined miRs	miR-1915-3p & miR-3692-3p				0.923	0.814	1.000	<0.001
		miR-1915-3p, -3692, -5001-5p & -6716-5p				1.000	1.000	1.000	<0.001
AAHGS vs AAGS7	Single miR	miR-3692-3p	-0.115	0.860	0.530	0.719	0.531	0.907	0.045
AAHGS vs CALGS	Single miR	miR5001-	0.585	0.600	0.600	0.669	0.474	0.863	0.115
		miR-6716-	-0.650	0.600	0.850	0.751	0.564	0.939	0.024
	Combined	miR-5001-5p & miR-6716-5p				0.795	0.623	0.967	0.008
AALGS vs AAGS7	Single miR	miR-5189-	-0.158	0.870	0.670	0.760	0.584	0.936	0.015
		miR-1915-	1.705	0.710	0.600	0.752	0.575	0.930	0.021
		miR-5001-	0.895	0.930	0.800	0.862	0.709	1.000	0.001
		miR-6716-	-0.705	0.770	0.730	0.797	0.626	0.969	0.008
	Combined miRs	miR-5001-5p & miR-6716-5p				0.831	0.658	1.000	0.003
		miR-5001-5p, miR-5189 & miR-6716-5p				0.872	0.717	1.000	0.001
		miR-1915-3p, -5001-5p, -5189 & -6716-5p				0.889	0.742	1.000	0.001
AALGS vs CAHGS	Single miR	miR-1915-	0.995	0.500	0.860	0.681	0.474	0.889	0.103
		miR-3692-	-0.305	0.730	0.740	0.884	0.707	0.982	0.001
	Combined	miR-1915-3p & miR-3692-3p				0.985	0.949	1.000	<0.001
AALGS vs	Single	miR-3692-	-1.550	0.670	0.790	0.710	0.515	0.909	0.055
	Combined	miR-1915-3p & miR-3692				0.890	0.770	1.000	0.001
AALGS vs	Single	miR-3692-	-1.450	0.670	0.800	0.793	0.629	0.957	0.006
	Combined	miR-1915-3p & miR-3692				0.910	0.807	1.000	<0.001
AAGS7 vs CALGS	Single miR	miR-5001-	0.870	0.800	0.800	0.798	0.633	0.962	0.005
		miR-6716-	-0.705	0.730	0.770	0.815	0.649	0.983	0.005
	Combined	miR-5001-5p & miR-6716-5p				0.851	0.705	0.998	0.002
AAGS7 vs CAGS7	Single miR	miR-3692-	-0.080	0.530	0.930	0.751	0.576	0.927	0.019
		miR-6716-	-0.205	0.600	0.870	0.740	0.560	0.920	0.025
	Combined	miR-3692-3p & miR-6716-5p				0.747	0.568	0.926	0.021
AAGS7 vs CAHGS	Single miR	miR-3692-	-1.115	0.930	0.600	0.818	0.666	0.970	0.003
		miR-5001-	0.915	0.800	0.730	0.796	0.629	0.962	0.006
		miR-6716-	-0.910	0.870	0.670	0.789	0.625	0.953	0.007
	Combined	miR-3692-3p & miR-5001-5p				0.827	0.682	0.971	0.002
		miR-3692-3p, -5001-5p & -6716-				0.831	0.687	0.975	0.002

AAHGS: AA men with high Gleason score; CAHGS: Caucasian men with high Gleason score
AALGS: AA men with low Gleason score; CALGS: CA men with low Gleason score

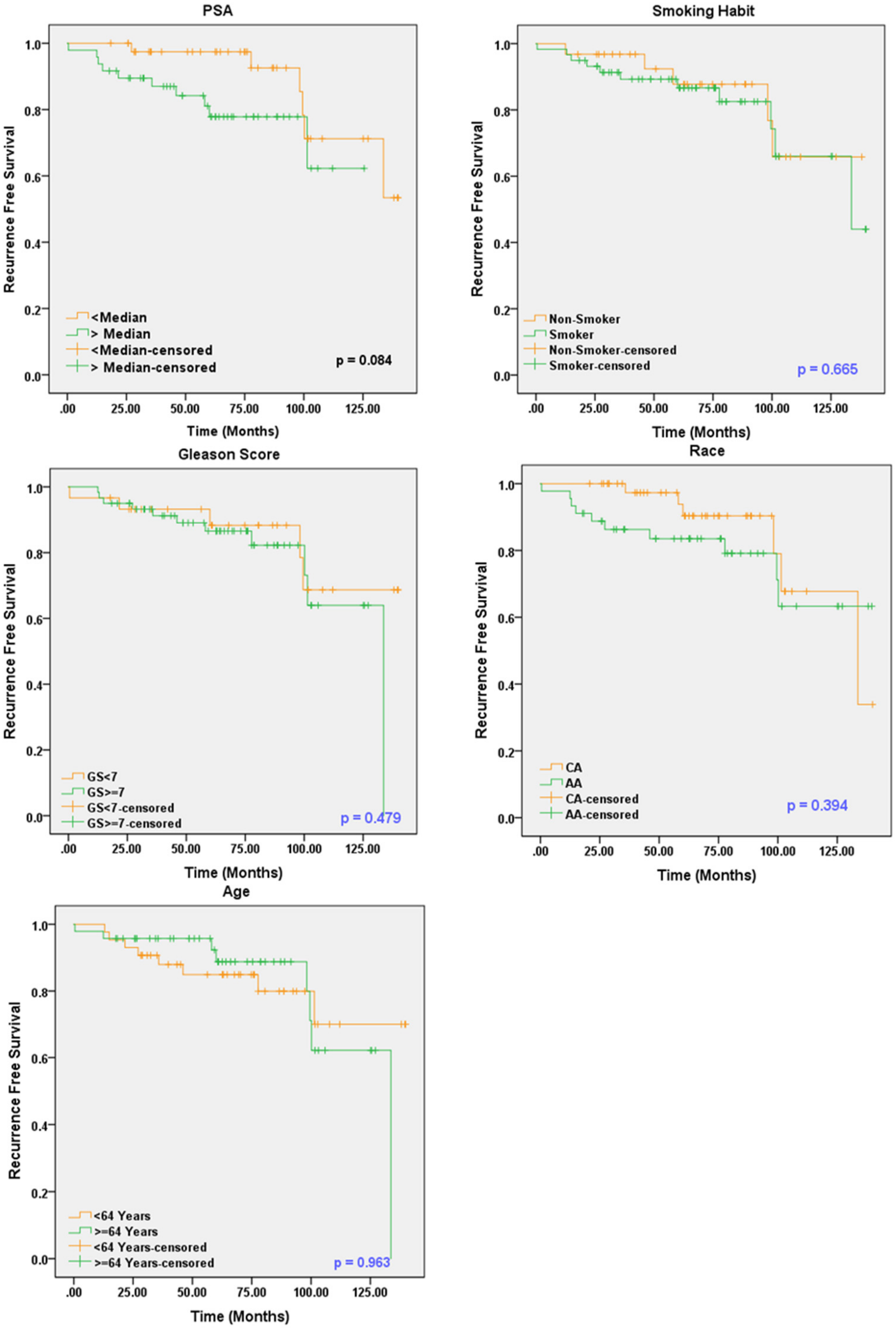
PSA
Recurrence Free Survival
<Median
> Median
<Median-censored
> Median-censored
p = 0.084
Time (Months)
Smoking Habit
Recurrence Free Survival
Non-Smoker
Smoker
Non-Smoker-censored
Smoker-censored
p = 0.665
Time (Months)
Gleason Score
Recurrence Free Survival
GS<7
GS>=7
GS<7-censored
GS>=7-censored
p = 0.479
Time (Months)
Race
Recurrence Free Survival
CA
AA
CA-censored
AA-censored
p = 0.394
Time (Months)
Age
Recurrence Free Survival
<64 Years
>=64 Years
<64 Years-censored
>=64 Years-censored
p = 0.963
Time (Months)

Figure S1. Expression of surface markers proteins in small extracellular (sEVs) vesicles derived from plasma of PCa patients. Western blot was performed on sEVs derived from plasma collected from AA and CA patients. The membrane was incubated with anti-CD63, CD9 and CD81antibodies. Plasma free from sEVs was used as a negative control. The developed signal was visualized at low and high exposure times.

Figure S2. Pathway prediction for small extracellular vesicle-associated miR-3201, miR-5001-5p, miR-6068, miR-6716-5p, miR-1915-3p, miR-3944-5p, miR-3939, miR-3692-3p, and miR-5189-5p. Pathway prediction was performed for sEV-associated miRs that can discriminate PCa patients from normal individuals (A), Gleason score (B) and race (C).

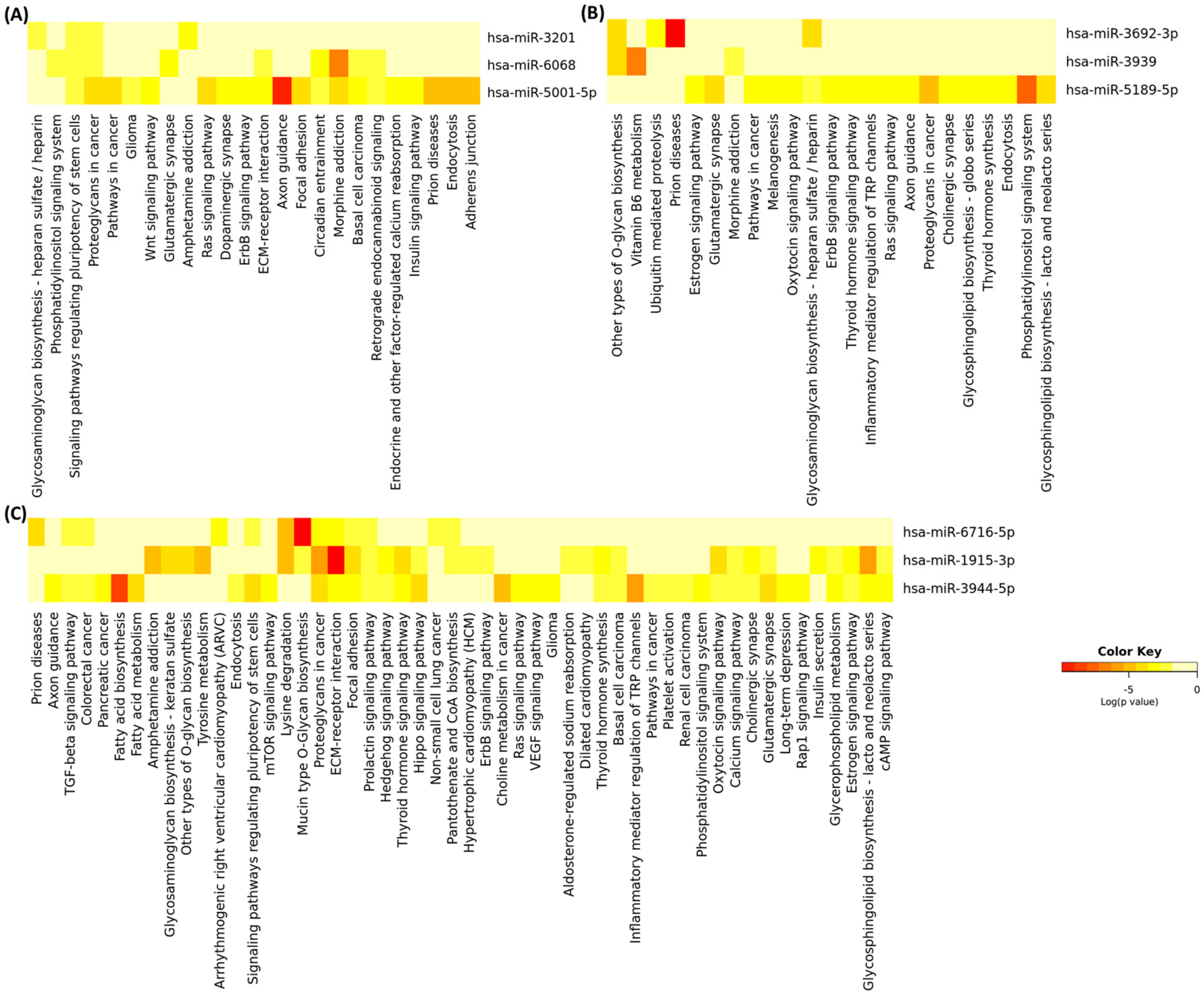

Figure S3: **Overall survival analysis for prostate cancer patients with different clinicopathological features**. Kaplan-Meier analysis shows no association between the tested parameters and the overall survival of PCa patients. The *p*-value was obtained by the log-rank test of the Kaplan-Meier curve.

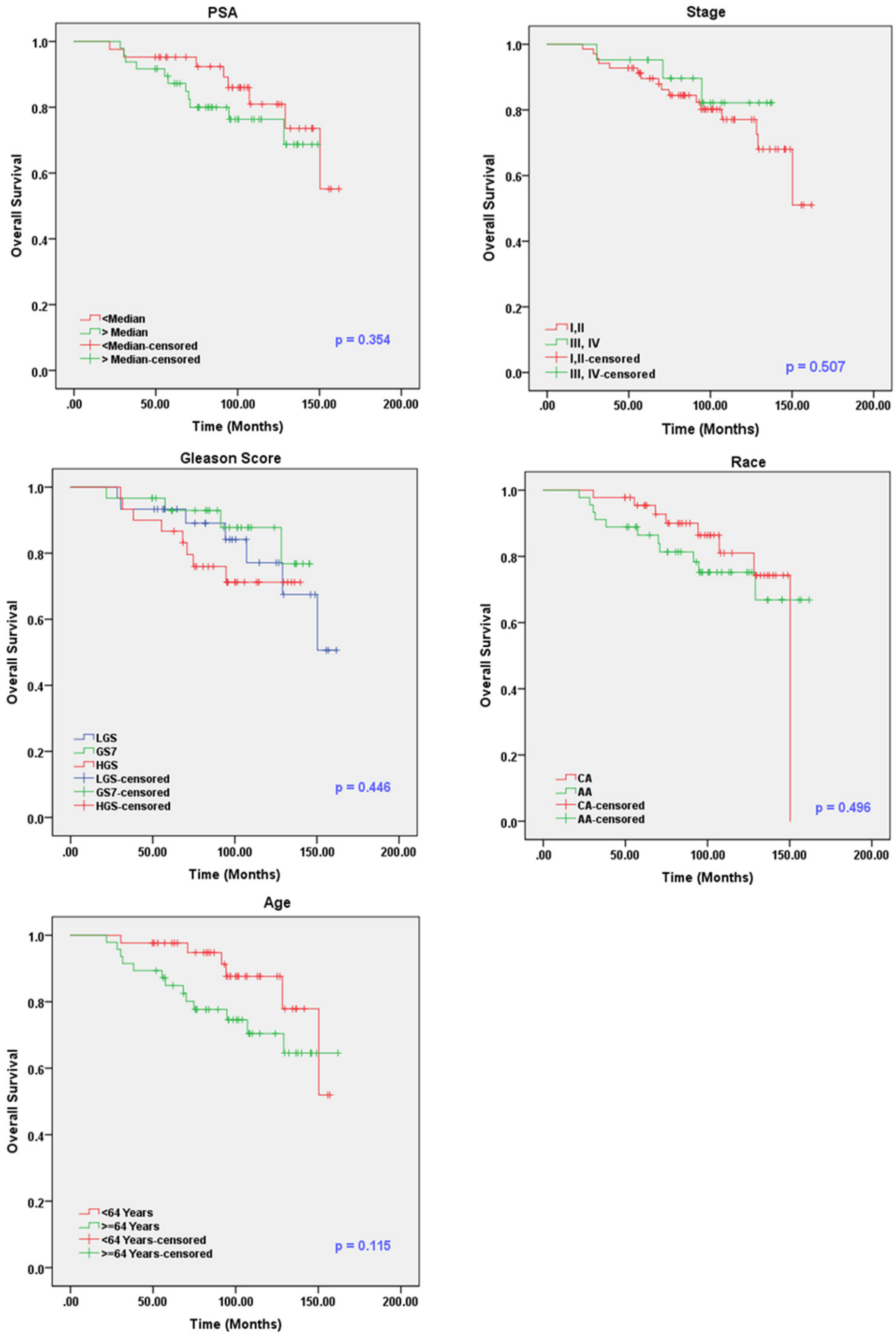

Figure S4. Recurrence-free survival analysis for prostate cancer patients with different clinicopathological features. Kaplan-Meier analysis shows no association between the tested parameters and the recurrence-free survival of PCa patients. The *p*-value was obtained by the log-rank test of the Kaplan-Meier curve.

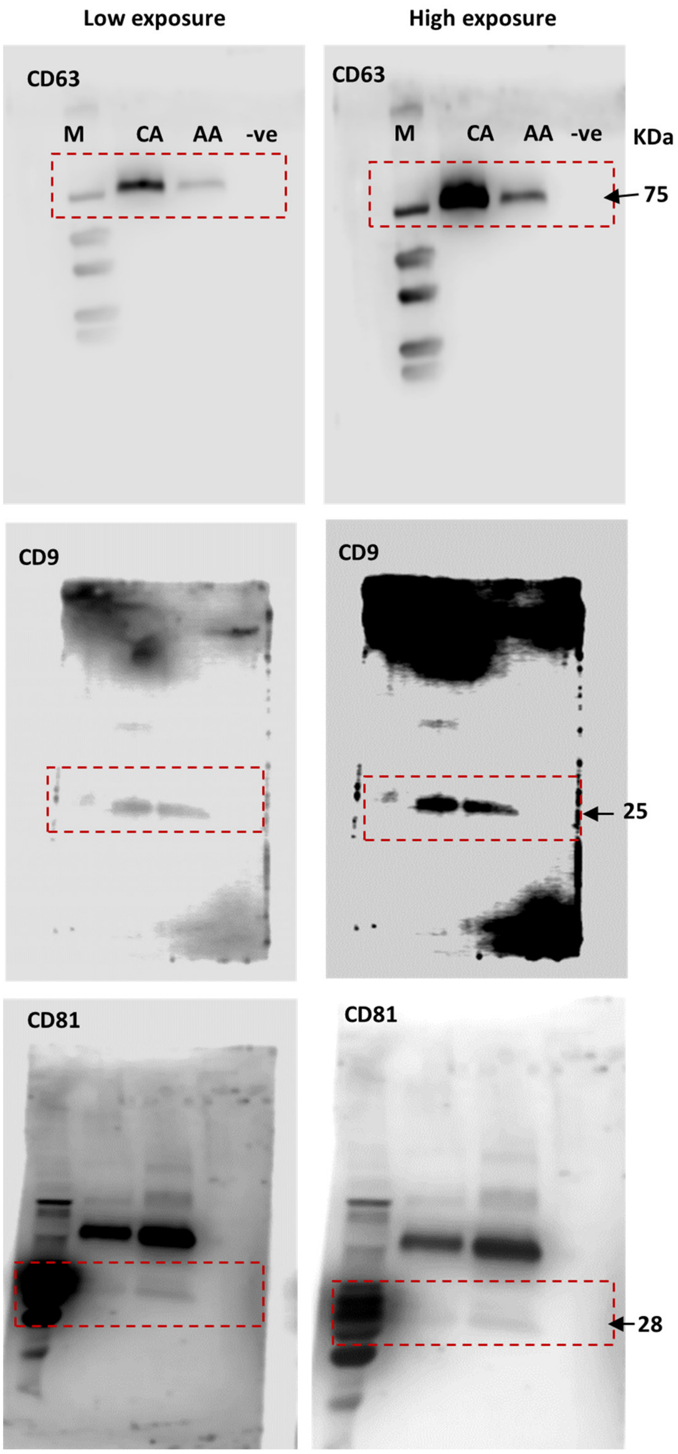

Supplement: Supplementary file 1 [file cancers-13-05236-s001.zip › cancers-1425791-supplementary.pdf]
